# The Tuberculosis Drug Candidate SQ109 and Its Analogs Have Multistage Activity against *Plasmodium falciparum*

**DOI:** 10.1021/acsinfecdis.4c00461

**Published:** 2024-08-14

**Authors:** Savannah J. Watson, Mariëtte E. van der Watt, Anjo Theron, Janette Reader, Sizwe Tshabalala, Erica Erlank, Lizette L. Koekemoer, Mariska Naude, Marianna Stampolaki, Feyisola Adewole, Katie Sadowska, Pilar Pérez-Lozano, Andreea L. Turcu, Santiago Vázquez, Jihee Ko, Ben Mazurek, Davinder Singh, Satish R. Malwal, Mathew Njoroge, Kelly Chibale, Oluseye K. Onajole, Antonios Kolocouris, Eric Oldfield, Lyn-Marié Birkholtz

**Affiliations:** ^†^Department of Biochemistry, Genetics and Microbiology, and ^‡^School of Health Systems and Public Health (SHSPH), University of Pretoria Institute for Sustainable Malaria Control, University of Pretoria, Hatfield, Pretoria 0002, South Africa; § Next Generation Health, Council for Scientific and Industrial Research, Pretoria 0001, South Africa; ∥ Centre for Emerging Zoonotic and Parasitic Diseases, National Institute for Communicable Diseases of the National Health Laboratory Services, Wits Research Institute for Malaria, Faculty of Health Sciences, University of the Witwatersrand, Johannesburg, Johannesburg 2000, South Africa; ⊥ Laboratory of Medicinal Chemistry, Section of Pharmaceutical Chemistry, Department of Pharmacy, National and Kapodistrian University of Athens, Panepistimioupolis-Zografou, 15771 Athens, Greece; # Department of Biological, Physical and Health Sciences, College of Science, Health & Pharmacy, Roosevelt University, 425 South Wabash Avenue, Chicago, Illinois 60605, United States; ∇ Department of Pharmacy and Pharmaceutical Technology and Physical Chemistry, Faculty of Pharmacy and Food Sciences, University of Barcelona, Barcelona 08028, Spain; ○ Laboratori de Química Farmacèutica (Unitat Associada al CSIC), Departament de Farmacologia, Toxicologia i Química Terapèutica, Facultat de Farmàcia i Ciències de l’Alimentació, and Institute of Biomedicine (IBUB), Universitat de Barcelona, Barcelona E-08028, Spain; ◆ Department of Chemistry, University of Illinois at Urbana-Champaign, Urbana, Illinois 61801, United States; ¶ Drug Discovery and Development Centre (H3D), University of Cape Town, Rondebosch, Capetown 7701, South Africa; †† South African Medical Research Council Drug Discovery and Development Centre, Department of Chemistry and Institute of Infectious Disease and Molecular Medicine, University of Cape Town, Rondebosch, Capetown 7701, South Africa

**Keywords:** Plasmodium falciparum, SQ109, antimalarial, transmission-blocking, multistage

## Abstract

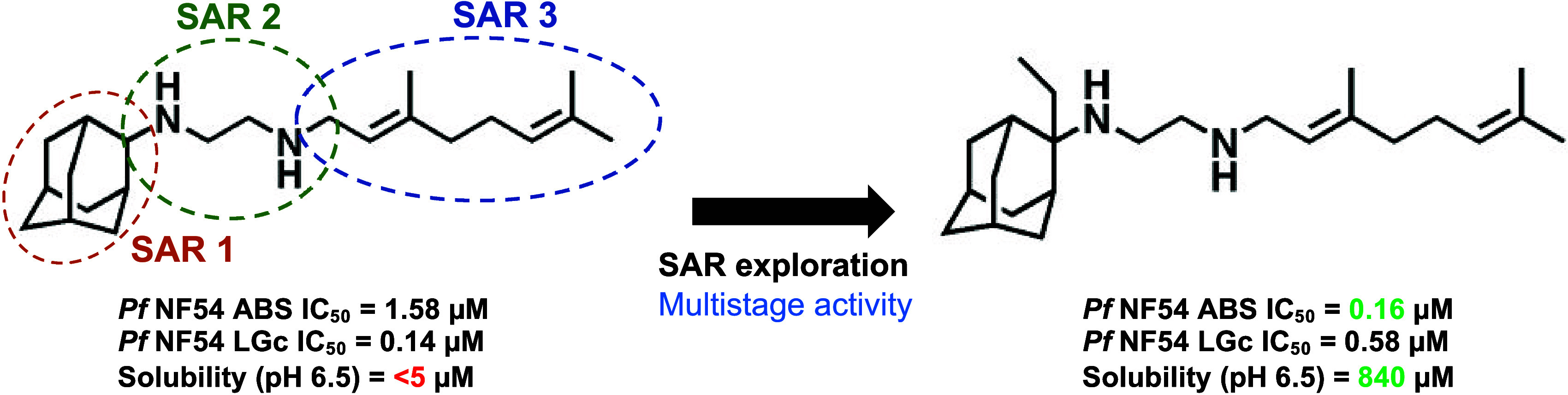

Toward repositioning the antitubercular clinical candidate SQ109 as an antimalarial, analogs were investigated for structure–activity relationships for activity against asexual blood stages of the human malaria parasite *Plasmodium falciparum* pathogenic forms, as well as transmissible, sexual stage gametocytes. We show that equipotent activity (IC_50_) in the 100–300 nM range could be attained for both asexual and sexual stages, with the activity of most compounds retained against a multidrug-resistant strain. The multistage activity profile relies on high lipophilicity ascribed to the adamantane headgroup, and antiplasmodial activity is critically dependent on the diamine linker. Frontrunner compounds showed conserved activity against genetically diverse southern African clinical isolates. We additionally validated that this series could block transmission to mosquitoes, marking these compounds as novel chemotypes with multistage antiplasmodial activity.

Malaria presents an immense challenge to governments and healthcare systems, particularly in tropical regions of the world, with an estimated 247 million cases and 619 000 deaths globally in 2021.^1^ The incidence of malaria is spread disproportionately and is primarily accounted for the African region by the World Health Organization (WHO) as a result of the prevalence of *Plasmodium falciparum*, the deadliest of all species, combined with the abundance of the *Anopheles* spp. mosquito vectors. The complexity of the *P. falciparum* (*Pf*) life cycle is underpinned by the various developmental stages within different niches in the human host and mosquito vector.^2^ Elimination of the disease, therefore, requires effective targeting of these multiple stages,^3,4^ including eliminating the asexual replicative stages (asexual blood stages (ABS)) and targeting parasite population bottlenecks that appear at the transition phases of the parasite life cycle to ensure parasite reproduction.^5^ Human-to-mosquito parasite transmission requires mature stage V gametocytes, which are formed by differentiation of gametocytes through five distinct morphological stages, where immature stages (stage I to IV) sequester in the bone marrow, but stage V gametocytes circulate in the peripheral blood system.^6^ Their developmental timeline and low blood circulation numbers make these stages amenable to pharmacological intervention.^7^ As per the target candidate profiles (TCPs; defined by the Medicines for Malaria Venture, www.mmv.org), compounds with activity against ABS parasites (TCP-1) as well as transmission-blocking ability (TCP-5)^2^ will have the potential to reduce parasite prevalence in endemic areas, clear asymptomatic individuals harboring parasites, and lower the spread of resistance that typically develops against compounds during asexual replication.^4,6,8^

The search for new antimalarial compounds with the ability to target ABS parasites and gametocytes has generally been biased toward compounds targeting the biology important to the asexual stages, with screening campaigns prioritizing hits based on ABS activity and gametocyte activity, adding dual activity value to these.^3^ An alternative approach previously reported *de novo*, parallel screening against multiple stages, which identified new chemical matter that can target specific life cycle stages of *Pf*.^4^ Interestingly, several compounds were identified with preferential activity against *Pf* late-stage gametocytes (LGc),^4^ providing new chemical starting points for further development and optimization as multistage active compounds. One such compound was the well-characterized antitubercular clinical candidate *N*-geranyl-*N*′-(2-adamantyl)ethane-1,2-diamine, SQ109/**1** (Figure 1). This compound displayed >10-fold selectivity toward LGc *in vitro* (IC_50_ = 0.14 μM) compared to ABS (IC_50_ = 1.58 μM),^5^ and its transmission-blocking ability was confirmed by its ability to target male gametes with a transmission-reducing activity at 80%.^4^ We show here that this activity extends to immature stage gametocytes (IGc, IC_50_ = 0.38 μM *vs* LGc IC_50_ = 0.14 μM) (Figure 1A).

**Figure 1:**
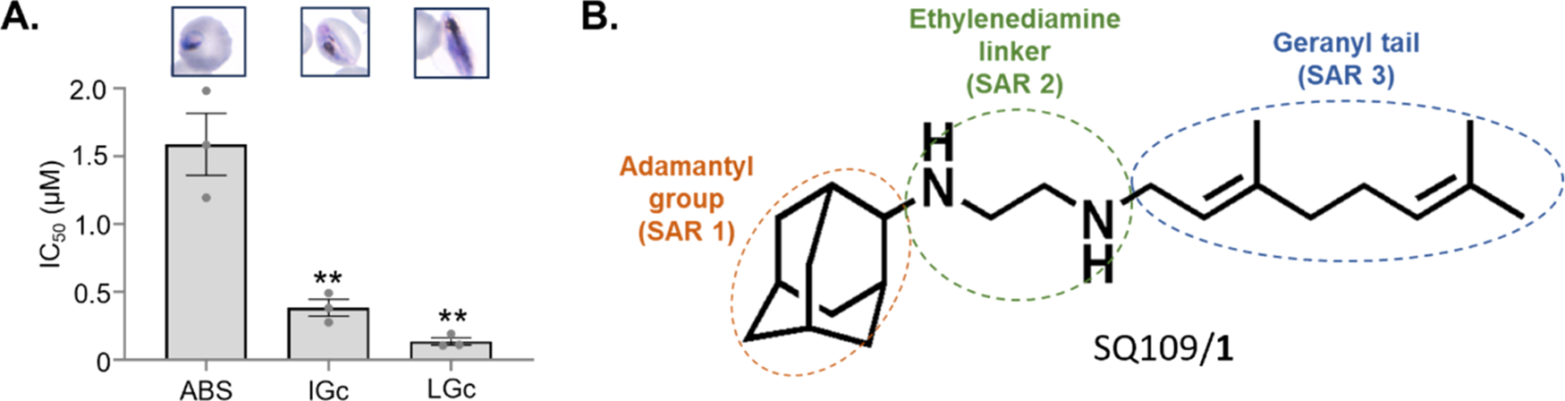
SQ109 is a gametocyte-targeting compound. (A) IC_50_ for parent compound SQ109/**1** on asexual parasites (ABS), immature-stage gametocytes (IGc, stage II/III), or late-stage gametocytes (LGc, stage IV/V), under 48 h drug pressure. IC_50_ ABS: 1.58 ± 0.2 μM; IC_50_ IGc: 0.38 ± 0.10 μM; IC_50_ LGc: 0.14 ± 0.03 μM; ** *P* < 0.01 indicates IGc and LGc *vs* ABS. (B) Chemical structure and summary of the designed structure–activity relationships (SAR-1, SAR-2 and SAR-3) around SQ109/**1**. Data are from >2 independent biological repeats, mean ± SE indicated, unpaired Student’s *t*-test applied.

SQ109/**1** is a second-generation ethylenediamine antitubercular that completed phase IIb clinical trials for tuberculosis, and potently targets multidrug-resistant *Mycobacterium tuberculosis* (*Mtb*).^6,7^ Its microbial target was identified as mycobacterial membrane protein large 3 (MmpL3),^8^ involved in cell wall biosynthesis. Additionally, SQ109 was also implicated in the disruption of the proton motive force (PMF)^9^ by acting as an uncoupler, and this has been proposed to be a significant driver of its activity against non-MmpL3 containing organisms, such as *Trypanosoma cruzi* and *Leishmania* spp,^10,11^ where effects on Ca^2+^ homeostasis are also found due to targeting of acidocalcisomes. Given its activity against multiple infectious organisms, a series of SQ109/**1** analogs with structural changes centered around the adamantyl C-2 position were evaluated for enhanced activity against mycobacteria and other bacteria and protists, including ABS *Pf*.^5^ We showed that analogs with ethyl, butyl, phenyl and benzyl substituents displayed improved ABS activity over SQ109, with minimal toxicity to human cells, making them of interest as potential multistage antimalarial leads.^5^

These results prompted further investigation of SQ109/**1** analogs, including their ability to target multiple parasite stages. Here, we evaluated the structure–activity relationships (SAR) of the SQ109/**1** scaffold as multistage active antiplasmodial compounds. SAR exploration involved alkyl and benzyl/phenyl substitution at the C-2 of the adamantyl or replacement of the entire adamantyl group with bioisosteres (SAR-1); methylation, extension, or replacement of the ethylenediamine linker (SAR-2) and modifications to the geranyl tail region (SAR-3) (Figure 1B).

## Results and Discussion

**Figure 2:**
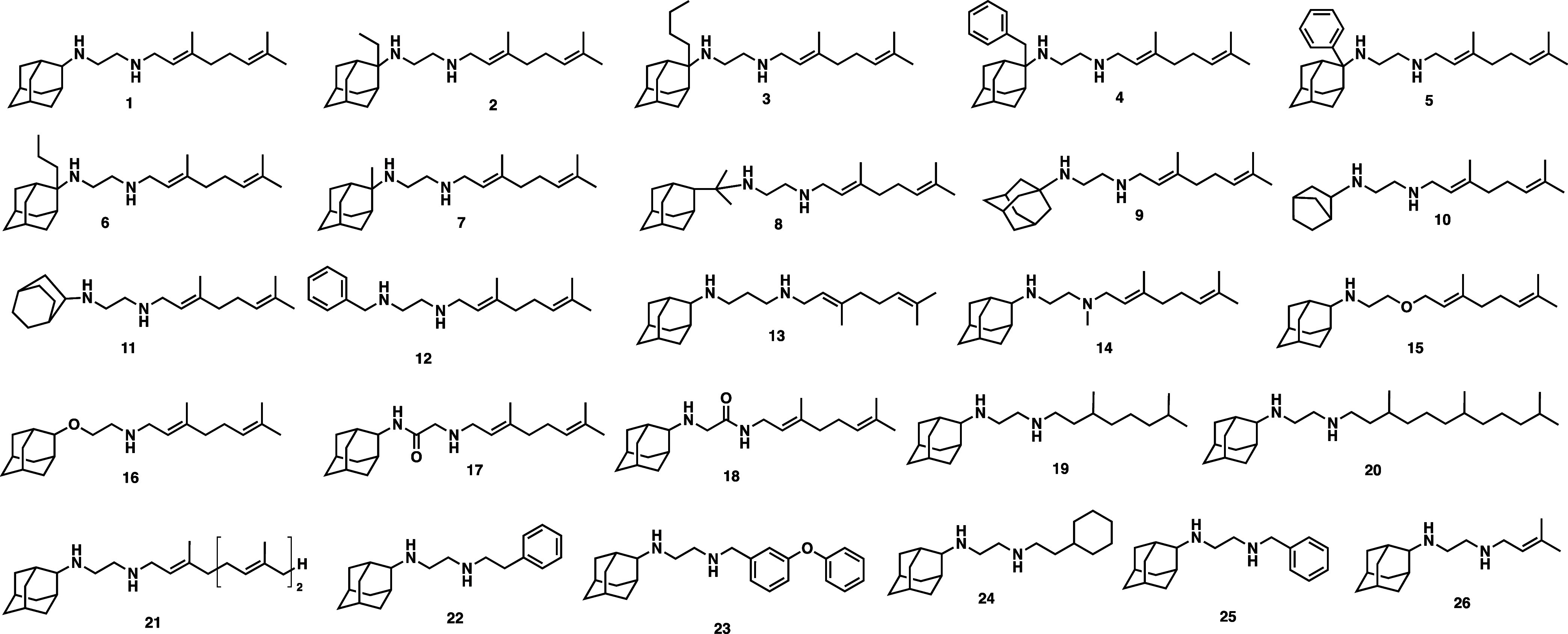
Structures of the compounds investigated. Compounds **1**-**12** are in SAR-1, **13**-**18** are in SAR-2, and **19**-**26** are in SAR-3.

We generated several analogs of SQ109/**1** (**2**–**26**, Figure 2) to explore the activity around SAR-1–3 (Figure 1). The synthesis of most compounds (**1**–**9**, **15**–**21**) has been described previously,^12,13^ but compounds **10**–**14** and **22**–**26** are new, and their synthesis is provided in the Supporting Information. The identity and purity of all compounds were verified by NMR, with >95% purity (Supporting Information Figures S3 and S4). All compounds were evaluated for their biological activity against ABS parasites of the drug-sensitive NF54 strain of *Pf* (*Pf*NF54 ABS) by detecting SYBR Green I fluorescence as an indicator of parasite proliferation,^14^ and compounds with IC_50_ < 1 μM were further profiled against the multidrug-resistant strain, *Pf*K1, to assess the potential for cross-resistance. Furthermore, compounds were evaluated against late (stage IV/V) gametocytes from *Pf*NF54 (*Pf*NF54 LGc), with viability detected with PrestoBlue,^15^ and orthogonally evaluated for toxicity against a human hepatocellular carcinoma line, HepG2.^16^ SQ109/**1** and selected analogs were also profiled against circulating clinical isolates of southern African origin to assess whether this series would be clinically and geographically relevant.

### Activity in SAR-1

Substitution at the adamantyl C-2 with alkyl or aryl groups led to improvements in ABS activity over compound **1**. For example, **2**–**5** displayed activity <0.5 μM, compound **2** (with the ethyl substitution) being 10-fold more active against ABS parasites than **1**, while maintaining activity against late-stage gametocytes (Table 1). These compounds retained their activity against drug-resistant parasites (*Pf*K1) and were not cytotoxic to HepG2 cells (≥50% viability@ 20 μM or IC_50_ > 25 μM). The compounds’ profile shifted to now include additional activity against ABS parasites along with LGc activity. This shift is significant and depends on the presence of the adamantyl group. Interestingly, “equipotency” (<2-fold change in IC_50_) against both ABS parasites and gametocytes could be attained with compounds **3**–**6**. However, replacement of the adamantyl with a phenyl group, as in **12**, or substitutions with other heterocycles as in **10** and **11**resulted in a comparative loss of ABS activity (pronounced in **12**), while retaining the gametocyte-selective profile observed for **1**.

**Table 1:**
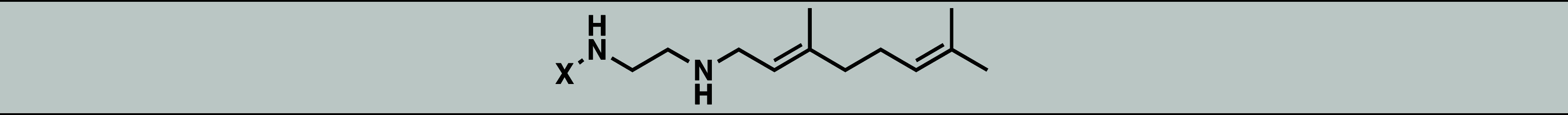

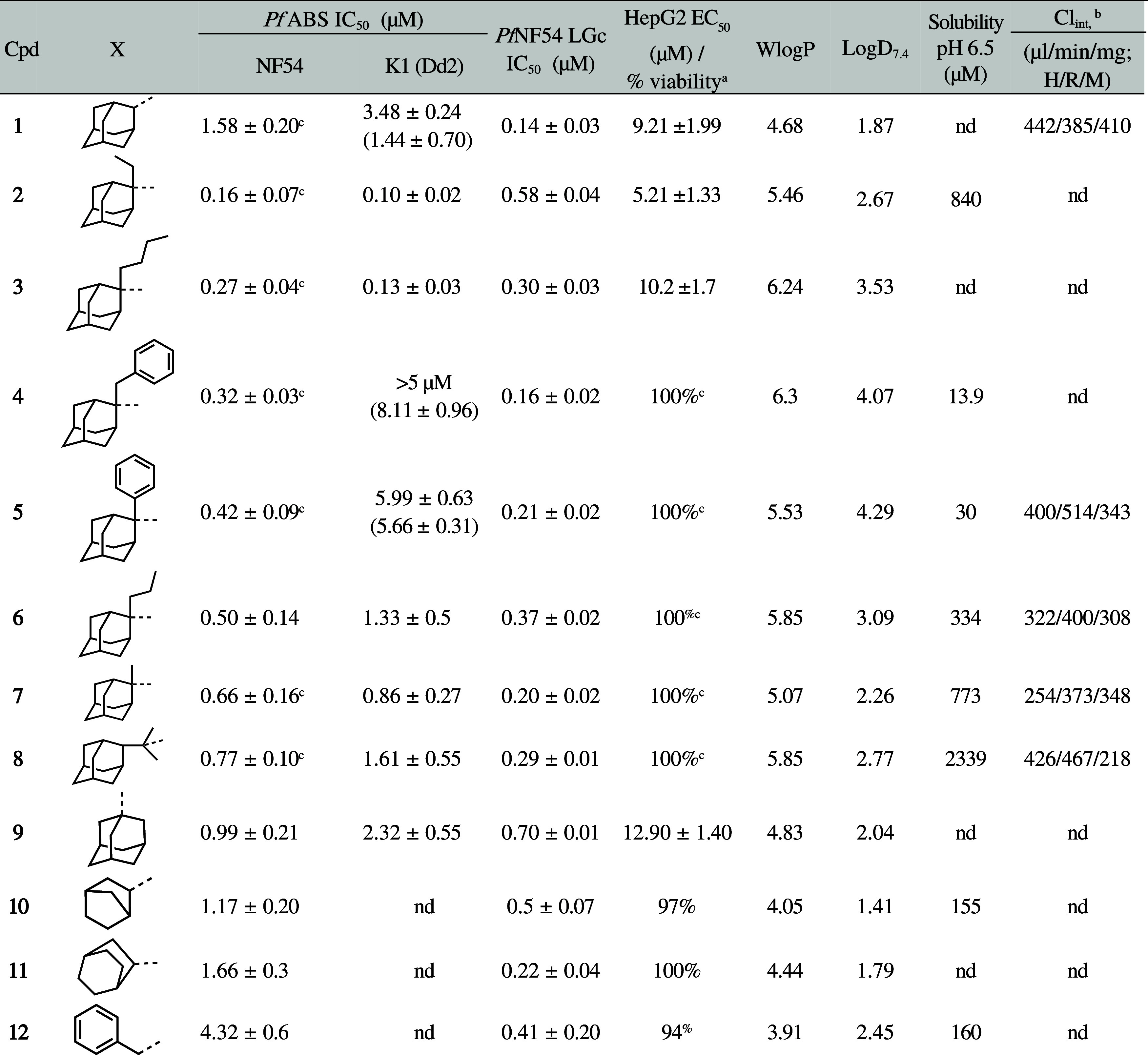
Antiplasmodial Activity of SQ109 Analogs with Changes around the Adamantane

a HepG2 viability as a % inhibition when treated with compound at 20 μM.

b Microsomal stability expressed as apparent intrinsic clearance (CL_int_) after 30 min incubation with human (H), rat (R) or mouse (M) liver microsomes (μL/min/mg).

c Published in Stampolaki *et al*.^5^ All cell growth inhibition data are from ≥2 independent biological repeats, each performed in technical duplicates, mean ± SE indicated. *W* log *P* was predicted using the free web tool, SwissADME^20^ and log *D*_7.4_ was computed using Chemicalize (htpps://chemicalize.com). nd = not determined.

The inclusion of the adamantyl group, therefore, seems to be a driver for the activity of these compounds against ABS parasites and is often used in drug design to provide lipophilicity without impeding solubility, moving a compound to a clinically useful log *P* space.^17^ Analysis of the solubility of these analogs showed that alkyl substitutions on the adamantyl somewhat improved solubility, with **2**, **6**, **7**, and **8** more soluble than **1**, **4**, and **5**. The introduction of the adamantyl group might also mean concomitant increased permeation of the blood-brain barrier,^17^ as observed for the azidothymidines used to treat HIV.^18^ Importantly, evaluation of lipophilicity using the calculated *W*log *P*(19,20) as well as log *D*_7.4_ (https://chemicalize.com) (Table 1) explained the activity shift of the analogs around SAR-1, with the more lipophilic analogs attaining activity against both ABS parasites and gametocytes, as we observed before for multistage antimalarials (Naude *et al*., submitted elsewhere). Indeed, lipophilicity was correlated with activity with *Pf*NF54 ABS for both *W* log *P* (Pearson *r* = −0.82, *P* = 0.0011, *n* = 12; Supporting Information Figure S1) and log *D*_7.4_ (to incorporate computed p*K*_a_ information additionally; Pearson *r* = −0.60, *P* = 0.039, *n* = 12; Supporting Information Figure S2). However, less lipophilic compounds **10**, **11**, and **12** only retain LGc activity, even though parasite-induced uptake mechanisms like the new permeability pathways (NPPs, a *Plasmodium* surface anion-selective channel that allow promiscuous uptake of various solutes), are inactive in mature gametocytes compared to ABS parasites,^21^ and this could support a different mode of action of these compounds in gametocytes. The high lipophilicity (>5) could be of concern regarding these compounds’ metabolic stability and oral bioavailability. However, the sterically bulky group has been shown to modulate intramolecular reactivity and restrict the access of hydrolytic enzymes, increasing drug stability and plasma half-life.^17^ We show that metabolic stability in liver microsomes is a concern for these compounds (Table 1), with only minor improvements seen against human microsomes for **7** and **8**, compared to **1**. The inclusion of the adamantyl group in antimalarials has been seen as critical to compounds such as the ozonide, OZ277 (Arterolane, approved as combination therapy with piperaquine; Synriam)^22^ and its next-generation analogs Artefenomel (OZ439), both of which present potent multistage activity, and where metabolic stability issues have been resolved. Further optimization of SQ109 analogs will need to critically address microsomal stability liabilities.

All the compounds, except for **4** and **5**, show comparable activity against the drug-resistant strain *Pf*K1. Interestingly, **4** and **5** are the only aromatic species in SAR-1 (**4** contains a benzyl substituent while **5** contains a phenyl group), with 16× and 14× higher IC_50_s against *Pf*K1. This strain is highly resistant to chloroquine and other antimalarials and is associated with increased export of *e.g.*, chloroquine from the digestive vacuole.^23^ This phenotype was also replicated in *Pf*Dd2 parasites, also resistant to chloroquine (among other antimalarials), with **4** and **5** losing activity but **1** similarly active (Table 1). These aromatic compounds may therefore be subject to resistance mechanisms similar to those found with chloroquine.

### Activity in SAR-2

Changes around the ethylenediamine linker in SAR-2 included an extension of the linker (propylenediamine; **13**), methyl substitution of the distal amine (**14**), and replacing either amine with an electron-withdrawing group (**15** and **16**, oxygen; **17** and **18**, carbonyl). Most of these changes were not tolerated for antiplasmodial activity and resulted in compounds with either similar ABS parasite activity to **1** (∼1.6 μM) or worse. This was even more dramatic for activity against late-stage gametocytes, where a 2- to >10-fold loss in activity was evident (Table 2), with gametocytocidal activity critically dependent on the diamine moiety. The loss of activity observed after the replacement of an amine suggests that both basic centers are required for activity against LGc, but not ABS (Table 2). This contrasts with the SAR associated with activity toward *Mtb* where only a single basic center is sufficient to maintain activity.^9^ Interestingly, compared to **1, 13** displayed 1.6-fold improved ABS (*Pf*NF54 ABS IC_50_ = 0.98 μM *vs* 1.58 μM for **1**) as well as 1.2-fold improved gametocyte activity (*Pf*NF54 LGc IC_50_ = 0.12 μM *vs* 0.14 μM), suggesting that extending the length of the linker carbon chain is tolerated, as was seen with a similar propanolamine^9^ against *Mycobacterium smegmatis* and *Pf*. As with the ethylenediamines, this activity might rely on the conservation of the basic centers and is reminiscent of the effect of other di- and polyamines on the parasite.^14^

**Table 2:**
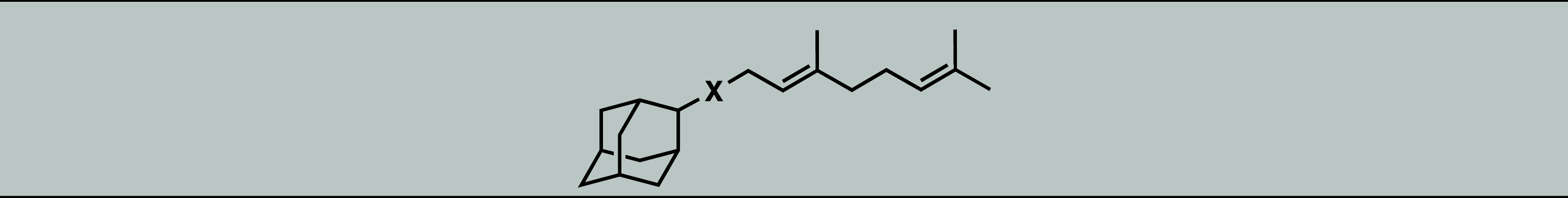

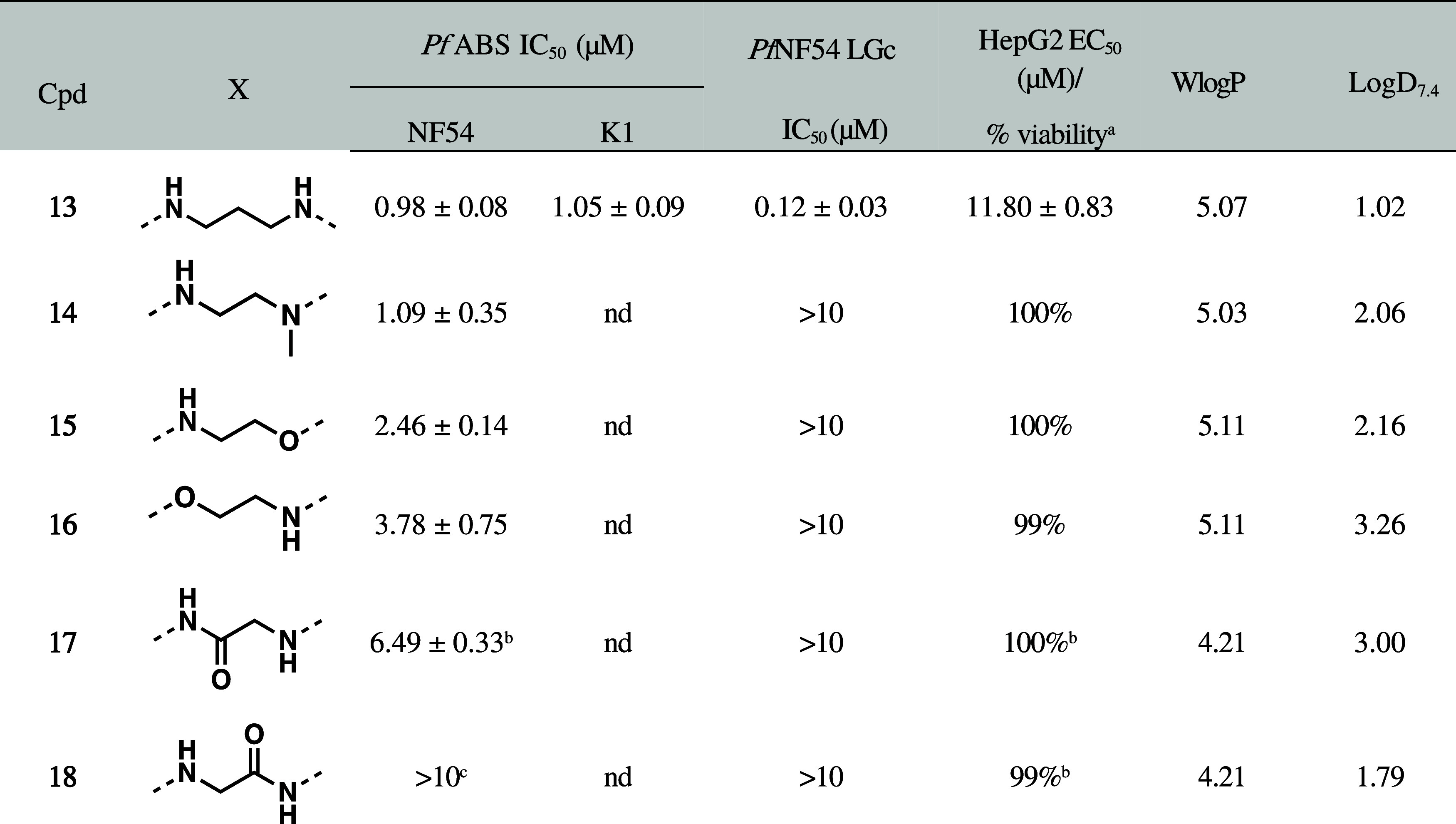
Antiplasmodial Activity of SQ109 Analogs with Changes around the Ethylenediamine Linker

a HepG2 viability as a % inhibition when treated with compound at 20 μM.

b Published in Stampolaki et al.^5^ All cell growth inhibition data are from ≥2 independent biological repeats, each performed in technical duplicates, mean ± SE indicated. WlogP was predicted using the free web tool, SwissADME^20^ and log *D*_7.4_ was computed using Chemicalize (https://chemicalize.com). nd = not determined.

### Activity in SAR-3

For SAR-3, replacing the geranyl tail with different aryl groups (**22**, **25** and **26**) maintained gametocyte activity while improving ABS activity. Extended (**21**; *Pf*NF54 ABS IC_50_ = 0.65 μM, *Pf*NF54 LGc IC_50_ = 0.51 μM) and/or saturated (**19**; *Pf*NF54 ABS IC_50_ = 0.20 μM, *Pf*NF54 LGc IC_50_ = 0.30 μM and **20**; *Pf*NF54 ABS IC_50_ = 0.39 μM, *Pf*NF54 LGc IC_50_ = 0.54 μM) tails were well tolerated, with these compounds also showing a promising shift to multistage activity. Previously, a similar observation was made for **21**, showing 2-fold improvement in activity against *Mtb*, compared to **1**. Other unsaturated analogs also maintained activity, however, saturation here led to a loss in antitubercular activity.^12^ Shortening the geranyl tail (**26**) retained gametocyte activity (*Pf*NF54 LGc IC_50_ = 0.37 μM) but with a loss in ABS activity (*Pf*NF54 ABS IC_50_ = 4.28 μM). However, the introduction of bulky groups led to a loss in gametocyte activity with a saturated ring not tolerated at all (**24**, IC_50_ = >10 μM against LGc), compared to only a 7-fold loss in the phenyl analog **22** (IC_50_ = 0.74 μM, Table 3). Aromatic groups were tolerated for ABS activity in **22** and **23**, but not when the tail length was further reduced as in **25**. Compounds **19**, **20** and **22** with saturated tails and compound **23** bearing a diphenyl ether moiety were moderately cytotoxic to HepG2 cells. The antiplasmodial activity for these SAR-3 analogs seems driven by lipophilicity, particularly for compounds **20** and **21**. As for SAR-1, ABS activity is lost for more hydrophilic analogs such as **24**-**26**.

**Table 3:**
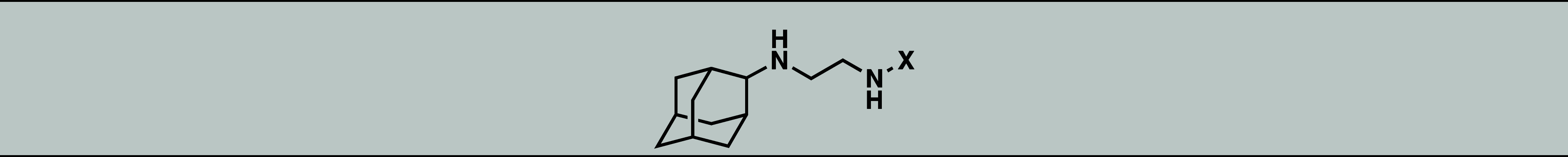

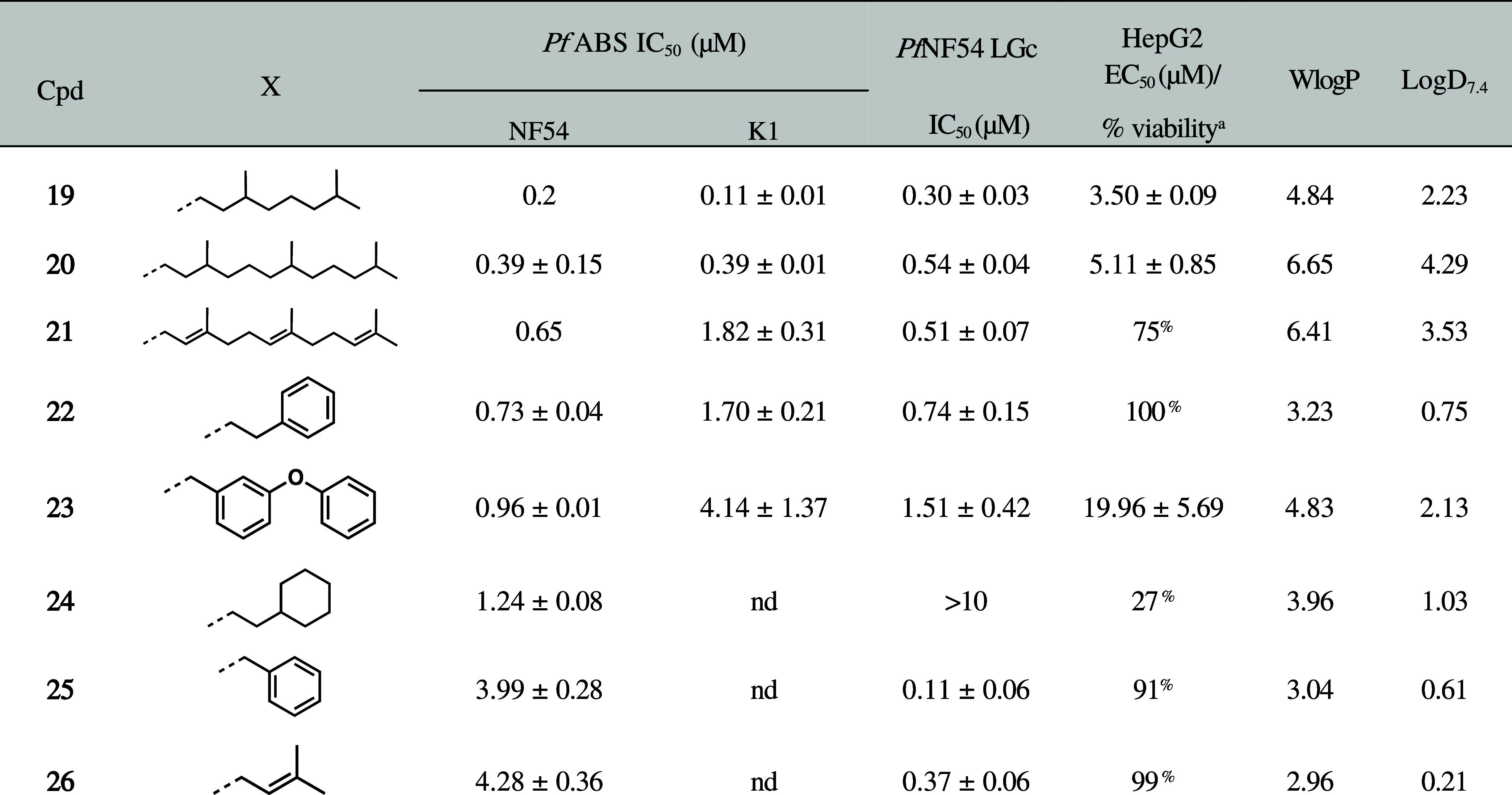
Antiplasmodial Activity of SQ109 Analogs with Changes around the Geranyl Tail

a HepG2 viability as a % inhibition when treated with compound at 20 μM. All cell growth inhibition data are from ≥2 independent biological repeats, each performed in technical duplicates, mean ± SE indicated. *W* log *P* was predicted using the free web tool, SwissADME^20^ and log *D*_7.4_ was computed using Chemicalize (htpps://chemicalize.com). nd = not determined.

All 25 analogs with ABS IC_50_ < 10 μM showed a good correlation (*r* = −0.64, *P* = 0.00051, Figure S1) between lipophilicity (*W* log *P*) and activity, which holds somewhat true for log *D*_7.4_ (Figure S2). A search of 633 computed descriptors (http://www.scbdd.com/chemdes/) did not improve correlations over those found with *W* log *P*. It is of interest that there is no correlation overall between gametocytocidal activity and *W* log *P* (*r* = 0.022, n = 20, *P* = 0.93, Figure S1) or with log *D*_7.4_ (*r* = 0.063, *n* = 20, *P* = 0.79, Figure S2), with high ligand-lipophilicity efficiency (LLE) obtained with potent gametocytocidal compounds (Figure S3), a relationship that was not as evident for the LLE for ABS activity. This would support different uptake mechanisms in ABS and mature gametocytes as suggested before (Naude *et al*., submitted elsewhere).^21^ However, the mechanisms of action (uncoupling activity, protein targets, Ca^2+^ homeostasis, *etc*.) could also be very different between these stages and cannot be ignored as explanations for the difference in activity. For example, in earlier work, it was shown that SQ109 targets Ca^2+^ homeostasis in other parasitic protozoa,^10,11^ targeting acidocalcisomes, and the digestive vacuole in malaria parasites has many similarities to these vacuoles,^22^ including, *e.g.*, a V-type H^+^-ATPase, previously proposed as one target for SQ109 in malaria parasites.^4^ Since the digestive vacuole does not play a major role in gametocytes, this could be one reason for the lack of correlation with lipophilicity, but more work on mechanisms is warranted.

### Transmission-blocking Potential of SQ109 and Its Analogs

To validate the transmission-blocking potential of SQ109 and the analogs, compounds with submicromolar activity against LGc were evaluated for their ability to prevent the formation of male and female gametes and oocysts, stages of the parasite that develop within the mosquito vector after gametocyte transmission from humans (Table 4). Most compounds showed comparable or improved activities to those found in **1** against both male and female gametes, but in 9 out of 14 cases, there was more activity against male gametes.

**Table 4:** Additional *In Vitro* Life Cycle Stage Profiling of the Most Potent Derivatives

			oocyst formation (% inhibition)	
Cpd	male gamete inhibition %	female gamete inhibition %	TRA^a^	TBA^b^
**1**	72.98 ± 5.97^c^	51.74 ± 5.40^c^	76.0 ± 4.75^c^	36.0 ± 10.6^c^
**2**	95.45 ± 4.55	78.88 ± 4.46	nd	nd
**3**	81.36 ± 8.64	67.05 ± 0.39	11	36
**4**	43.94 ± 10.61	74.48 ± 2.26	nd	26
**5**	48.01 ± 24.82	61.03 ± 14.35	38	63
**6**	34.59 ± 14.27	64.59 ± 12.33	53	67
**7**	27.14 ± 14.02	34.05 ± 9.23	nd	nd
**8**	23.51 ± 9.72	54.00 ± 2.54	nd	nd
**9**	96.67 ± 3.33	90.50 ± 7.17	70	47
**13**	90.91 ± 9.09	87.02 ± 3.68	70	64
**19**	95.45 ± 4.55	86.30 ± 2.58	47	36
**20**	95.0 ± 5.00	77.52 ± 10.85	75	54
**21**	95.45 ± 4.55	86.30 ± 2.58	56	20
**22**	92.12 ± 1.21	85.40 ± 7.62	89	80

a Transmission-reducing activity (TRA) after a 48 h drug treatment at 2 μM, of a single biological repeat.

b Transmission-blocking activity (TBA) after a 48 h drug treatment at 2 μM, of a single biological repeat.

c Published in Reader et al.^4^ nd–not determined.

Compounds **19–22**, as well as **9** and **13**, all have activity (∼70–600 nM) against both male and female gametes, with the most potent activity (70–200 nM) against the male gametes (Table 4), similar to the majority of antimalarial candidates that also show preferential activity against male gametes.^24^ However, this sex-specificity is not found for **4**-**8**, which also have good activity against ABS parasites. This may point to a specific mode of action of these compounds in the parasite’s different stages constitutive to ABS parasites and female gametes and does not influence the explosive replication events required for male gamete formation.^25,26^ Gametocidal inhibition did not correlate with lipophilicity. Thus, while there is potent activity against both ABS and sexual forms, the strong lipophilicity correlation is only seen with the ABS forms, particularly with *Pf*NF54.

Transmission-blocking activity was confirmed in the standard membrane feeding assay (SMFA), by determining transmission-reducing activity (TRA; reduction in oocyst counts) and transmission-blocking activity (TBA, reduction in oocyst prevalence), 8–10 days after feeding female mosquitoes on gametocyte-infected blood, treated with selected compounds (at 2 μM). Most compounds reduced the TRA and TBA by >40% (Table 4), with changes in the geranyl tail again correlating with higher TBA and TRA.

**Figure 3:**
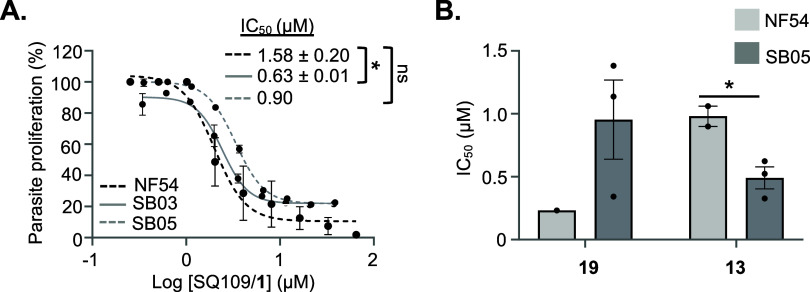
SQ109 displays *ex vivo* activity on African field isolates. (A) Dose–response analysis of SQ109 on clinical isolates SB03 and SB05 in comparison to lab-adapted NF54 *P. falciparum* parasites. Data are from at least 2 independent biological repeats, mean ± SE indicated, * *P* < 0.05 is indicated. (B) IC_50_ for SQ109 analogs **13** and **19** against a contemporary African field isolate SB05. IC_50_ of **19** on NF54: 0.2 *vs* on SB05: 0.95 ± 0.31 μM; IC_50_ of **13** on NF54: 0.98 ± 0.08 *μM vs* on SB5:0.49 ± 0.09 μM, * *P* < 0.05 is indicated.

The efficacy of **1** and its analogs, **13** and **19**, when evaluated against contemporary African clinical isolates *ex vivo*, was promising (Figure 3), with no significant shift in IC_50_ observed for 1 and only a ∼2-fold shift for analogs bearing a saturated tail and extended linker. This suggests potential clinical efficacy toward parasites from a geographically relevant, endemic region. A recent investigation into the link between parasite genetic complexity and differential efficacy of lead antimalarial agents against *Pf* clinical isolates suggests that antimalarials are active against both mono- and multiclonal ABS parasites but that this efficacy decreases toward gametocytes from genetically complex, multiclonal isolates.^27^ Since SB03/05 are both multiclonal,^27^ this warrants further investigation into the activity of this series toward clinical gametocytes.

## Conclusions

Here, we designed and synthesized analogs around the core of the antitubercular clinical candidate, SQ109, to achieve multistage antiplasmodial activity. Not only were we able to mimic the gametocytocidal activity of SQ109 previously observed, but we also saw a shift to equipotent dual-activity with marked improvements in ABS activity, suggesting that these compounds can be used both to inhibit ABS parasite proliferation as well as transmission of the sexual stages. This profile depends on the diamine linker, with various substitutions on the adamantane and changes to the geranyl tail tolerated. We postulate that this dual-activity is driven by lipophilicity due to the correlation with *W*log *P* and log *D*_7.4,_ particularly for ABS activity. This is supported by lipid diffusion as a potential main uptake mechanism, especially in gametocyte stages deficient in facilitated import mechanisms. Although high lipophilicity poses a caveat to this series’ metabolic stability and oral bioavailability, there is scope for improving its clinical relevance, as SQ109 has already been in several promising clinical trials for tuberculosis. While the adamantane headgroup affords the necessary lipophilicity and improves plasma half-life, further analogs increasing the polarity around the geranyl tail might be considered, as exemplified by the multistage active ozonide antimalarials. Solubility might be further modulated by minor changes to the linker while maintaining the cationic centers required for dual-activity.

## Methods

### Chemistry and ADME Assays

#### Synthesis and Purity Assessment

All synthetic procedures and characterizations are presented in the Supporting Information.

#### Solubility in 1% DMSO: 99% PBS Buffer

The stock solutions (10^–2^ M) of the assayed compounds were diluted to decreased molarity, from 300 to 0.1 μM, in 384 well transparent plate (Greiner 781801) with 1% DMSO:99% PBS buffer. Then, they were incubated at 37 °C and the light scattering was measured after 2 h in a NEPHELOstar Plus (BMG LABTECH). The results were adjusted to a segmented regression to obtain the maximum concentration in which compounds are soluble.

#### Aqueous Solubility

The solubility of each product was evaluated using an HPLC Agilent 1100 with a DAD detector. The samples were injected in an Agilent Poroshell 120 EC-C18, 2.7 μm, 50 mm × 4.6 mm column at 40 °C and 0.6 mL/min flow. The mobile phase was a mixture of A = water with 0.05% formic acid and B = acetonitrile with 0.05% formic acid. The procedure followed was from 95% A–5% B to 100% B in 3 min, 100% B in 3 min, from 100% B to 95% A–5% B in 1 min, 95% A–5% B in 3 min; the injection volume was set at 75 μL. To establish the solubility of the different compounds, a saturated solution of each sample in 2–5 mL buffer solution at pH 6.0 was prepared under stirring conditions at 37 °C. The samples remained at these conditions between 12–24 h. Then, the samples were filtered through a 0.45 μm PVDF filter and either directly analyzed or diluted before analyzing. The obtained area under the curve was compared with the one from the standard solution.

#### Microsomal Stability

The microsomal stability assay used a single-point assay design (Di et al., 2004). Briefly, the compounds were incubated at 1 μM in human, rat, and mouse liver microsomes (0.4 mg/mL) for 30 min at 37 °C. Reactions were quenched by adding ice-cold acetonitrile containing internal standard (carbamazepine, 0.0236 μg/mL). The samples were then centrifuged, and the samples were analyzed by liquid chromatography with tandem mass spectrometry (LC-MS/MS) (Agilent Rapid Resolution HPLC, AB SCIEX 4500 MS) for the disappearance of the parent compound. Half-life, clearance and hepatic excretion ratios were determined using standard equations.

### Biology: Culture Maintenance and *In Vitro* Assays

#### Ethics Statement

The *in vitro* work described holds ethics approval from the University of Pretoria (Health Sciences (506/2018) and Natural Sciences (180000094) Research Ethics Committee and the University of the Witwatersrand Human Research Ethics Committee (M130569) and Animal Ethics Committee (20190701–7O)).

#### *In Vitro* Cultivation of Asexual Blood Stages

*P. falciparum* asexual parasites were cultured from drug-sensitive NF54 (*Pf*NF54) and multidrug resistant strains *Pf*K1. Parasites were maintained in human erythrocytes (O+, suspended at 5% hematocrit) in RPMI-1640 culture medium (23.81 mM sodium bicarbonate, 0.024 mg/mL gentamycin, 25 mM HEPES pH 7.5, 0.2% d-glucose, 0.2 mM hypoxanthine and Albumax II) under hypoxic conditions (5% CO_2_, 5% O_2_ and 90% N_2_) with moderate shaking (60 rpm) at 37 °C. Cultures were synchronized to >95% in the ring stage with 5% (w/v) D-sorbitol treatment.^28^

#### *In Vitro* Cultivation of Gametocytes

Gametocytogenesis was induced on highly synchronized (>95%) asexual ring-stage parasites from *Pf*NF54 at 0.5% parasitemia and 6% hematocrit and thereafter maintained in glucose-deprived media as previously described.^29,15^ After 3 days, the hematocrit was decreased to 4%. Parasites were maintained in complete culture media supplemented with 50 mM *N*-acetyl-glucosamine 5–10 days post induction to inhibit proliferation of residual asexual blood stages. Gametocyte progression and morphology was also monitored microscopically using Giemsa-stained thin smears.

#### SYGR Green I Assay

Activity against the asexual stage of *P. falciparum* was evaluated using the SYBR Green I-based fluorescence assay as previously described.^4,16^ Cellular proliferation was measured on synchronized *in vitro Pf*NF54 ring-stage parasites (1% parasitemia, 2% hematocrit), following drug pressure for 96 h. Chloroquine was used as the positive drug control for complete inhibition of parasite proliferation.

#### PrestoBlue Fluorescence Assay

##### In vitro

 activity against the sexual stages of *P. falciparum* was evaluated using the PrestoBlue fluorescence assay as previously described.^4^ Activity was measured on *Pf*NF54 stage IV/V gametocytes (2% gametocytemia, 5% hematocrit) previously seeded with compounds (in non-lethal DMSO) for 48 h drug pressure, after which PrestoBlue reagent was added and incubated for 2 h. Fluorescence was detected in the supernatant at 612 nm. Dihydroartemisinin (DHA) was used as a positive drug control.

#### Male Gamete Exflagellation Inhibition Assay (EIA)

The EIA was performed using video microscopy by capturing the movement of exflagellation centers over time as previously described.^4,30^ Mature *Pf*NF54 stage V gametocytes (>95%;) were treated with 2 μM of drug for 48 h before the activation of exflagellation, without removing drug pressure. Movement was semi-automatically quantified from 15 videos of 8–10 s each between 15 and 22.5 min after incubation. Each video was analyzed using ICY-bio image analysis software.

#### Female Gamete Inhibition Assay

Similarly, female gametes were activated as described previously.^31^ Monoclonal anti-*Pfs* antibody conjugated to FITC was used to detect female gametes. Image acquisition was performed using a Zeiss Axio Lab.A1 epifluorescence microscope with a 100/1.4 numerical aperture (NA) oil immersion. Thirty images were captured per sample and analyzed manually.

#### Standard Membrane Feeding Assay (SMFA)

SMFA was performed using glass feeders as is outlined.^4,16^ Once again, mature stage V gametocytes (>95%) were treated with 2 μM of each compound for 48 h prior to feeding. *Anopheles coluzzii* females were allowed to feed on gametocyte cultures (1.5–2.5% gametocytemia, 50% hematocrit in A+ male serum with fresh erythrocytes) in the dark for 40 min. All unfed or partially fed mosquitoes were removed, and remaining females housed for 8–10 days. Mosquitoes were dissected to remove midguts, and oocysts counted under bright field illumination at ×20–×40 magnification. The percentage of block in transmission (transmission-blocking activity; TBA) and the percentage of reduction in the number of oocysts (transmission-reducing activity; TRA) were calculated after normalizing to the control untreated sample.

### Toxicity Assessment

#### *In Vitro* Activity against HepG2 Cells

Human hepatocellular liver carcinoma cells (HepG2) were cultivated *in vitro* in Dulbecco’s Modified Eagle’s Medium (DMEM) supplemented with 10% (v/v) heat inactivated fetal bovine serum and 1% (v/v) penicillin/streptomycin.^14^ Cells were detached with trypsin treatment at 80% confluency and were seeded in 96-well plates to adhere for 24 h under 5% CO_2_, humid (95%) conditions. Cells were subsequently exposed to drug pressure for 24 h and cytotoxicity was then determined using the lactate dehydrogenase (LDH) release assay through the colorimetric detection at 450 nm, as previously outlined.^4,16^

### Data Analysis and *In Silico* Predictions

Assay reproducibility was evaluated by *Z*′-factors and data are from a minimum of 3 independent biological repeats as indicated; statistical evaluation was performed with paired, two-tailed *t*-est (GraphPad Prism 8.3.0). The physicochemical properties of the compounds were predicted using either StarDrop version: 7.4.0.35635 (Optibrium Ltd.), the online web server, SwissADME,^32^ and at htpps://chemicalize.com.

## Supporting Information Available

Chemical synthesis and characterization; correlations between *Pf*ABS and late-stage gametocyte viability inhibition by SQ109 and its analogs (logIC_50_, μM) with W log *P* (Figure S1); correlations between *Pf*ABS and late-stage gametocyte viability inhibition by SQ109 and its analogs (logIC_50_, μM) with log *D*_7.4_ (Figure S2); PfABS and late-stage gametocyte (LGc) activity by SQ109 and its analogs associated to lipophilic ligand efficiency (LLE) (Figure S3). ^1^H and ^13^C NMR spectra for final compounds (Figure S4); qNMR spectra (Figure S5); compound SMILES (PDF)

## Abbreviations

SMFA: standard membrane feeding assay
TBA: transmission-blocking activity
TCP-1: target candidate profile-1
TCP-5: target candidate profile-5
TRA: transmission-reducing activity
