# Supplementary material for: The Tuberculosis Drug Candidate SQ109 and Its Analogs Have Multistage Activity against Plasmodium falciparum
Source: ACS Infect Dis. 2024 Aug 14;10(9):3358–67. doi: 10.1021/acsinfecdis.4c00461 (PMC11406516; doi:10.1021/acsinfecdis.4c00461)
Supplement: Supplementary file 1 — id4c00461_si_001.pdf [file id4c00461_si_001.pdf]

Supporting Information

The Tuberculosis Drug Candidate SQ109 and its Analogs Have Multi-Stage Activity Against *Plasmodium falciparum*

Savannah J. Watson[1], Mariëtte E. van der Watt[2], Anjo Theron[3], Janette Reader[1], Sizwe Tshabalala[1], Erica Erlank[4], Lizette L. Koekemoer[4], Mariska Naude[1], Marianna Stampolaki[5], Feyisola Adewole[6], Katie Sadowska[6], Pilar Pérez-Lozano[7], Andreea L. Turcu[8], Santiago Vázquez[8], Jihee Ko[9], Ben Mazurek[9], Davinder Singh[9], Satish R. Malwal[9], Mathew Njoroge[10], Kelly Chibale[10,11], Oluseye K. Onajole[6], Antonios Kolocouris[5], Eric Oldfield[9], and Lyn-Marié Birkholtz[1]

[1]Department of Biochemistry, Genetics and Microbiology, and [2]School of Health Systems and Public Health (SHSPH), University of Pretoria Institute for Sustainable Malaria Control, University of Pretoria, Hatfield 0002, South Africa

[3]Next Generation Health, Council for Scientific and Industrial Research, Pretoria 0001, South Africa

[4]Wits Research Institute for Malaria, Faculty of Health Sciences, University of the Witwatersrand, Centre for Emerging Zoonotic and Parasitic Diseases, National Institute for Communicable Diseases of the National Health Laboratory Services, Johannesburg

[5]Laboratory of Medicinal Chemistry, Section of Pharmaceutical Chemistry, Department of Pharmacy, National and Kapodistrian University of Athens, Panepistimioupolis−Zografou, 15771, Athens, Greece

[6]Department of Biological, Physical and Health Sciences, College of Science, Health & Pharmacy, Roosevelt University, 425 South Wabash Avenue, Chicago, IL 60605, United States.

[7]Department of Pharmacy and Pharmaceutical Technology and Physical Chemistry, Faculty of Pharmacy and Food Sciences, University of Barcelona, Barcelona, 08028, Spain

[8]Laboratori de Química Farmacèutica (Unitat Associada al CSIC), Departament de Farmacologia, Toxicologia i Química Terapèutica, Facultat de Farmàcia i Ciències de l'Alimentació, and Institute of Biomedicine (IBUB), Universitat de Barcelona, Barcelona, E-08028, Spain

[9]Department of Chemistry, University of Illinois at Urbana-Champaign, Urbana, Illinois 61801, United States

[10]Drug Discovery and Development Centre (H3D), University of Cape Town, Rondebosch 7701, South Africa

[11]South African Medical Research Council Drug Discovery and Development Centre, Department of Chemistry and Institute of Infectious Disease and Molecular Medicine, University of Cape Town, Rondebosch 7701, South Africa

*Correspondence:

Lyn-Marié Birkholtz

lynmarie.birkholtz@up.ac.za

Table of Contents:

1) Chemical synthesis and characterization
2) Supplementary Figure S1
3) Supplementary Figure S2
4) Supplementary Figure S3
5) ^{1}H and ^{13}C NMR spectra for final compounds (Figure S4)
6) qNMR spectra (Figure S5)
7) Compound SMILES

EXPERIMENTAL PROCEDURES

Chemistry

General information: All solvents and chemicals were used as purchased without further purification. The progress of all reactions was monitored using Merck silica gel 60 F254 plates mainly using chloroform/methanol/ammonia solutions as eluents. Norcamphor, 2-adamantanone, 3,3-dimethylallyl bromide, pyridinium chlorochromate and anhydrous methanol were purchased from Sigma-Aldrich; anhydrous ethylenediamine was purchased from TCI America; bicyclo[2.2.2]octan-2-one was purchased from Combi-Blocks; phenylacetaldehyde was purchased from Thermo Fisher Scientific; 3-phenoxybenzaldehyde was purchased from Ambeed Inc; 2-cyclohexylethanol was purchased from Alfa Aesar. ^{1}H NMR and ^{13}C NMR spectra were recorded on a Bruker spectrometer (300 and 75 MHz), with TMS as an internal standard. Standard abbreviations indicating multiplicity were used as follows: s = singlet, d = doublet, dd = doublet of doublets, t = triplet, q = quadruplet, m = multiplet and br = broad. The structures of **10-14** and **22-26** were determined using ^{1}H, ^{13}C and HRMS. ^{1}H and ^{13}C NMR spectra for the free amines were recorded in $CDCl_3$. HRMS experiments were performed on a micrOTOF-Q (Bruker) instrument while melting point data were collected using a Stuart Digital melting point apparatus (SMP30). qNMR data for new compounds were obtained at 600 MHz using a Bruker NEO spectrometer equipped with a Prodigy cryoprobe and a SampleXpress autosampler. Sample and trimethoxybenzene standard (TraceCERT®, Manufactured by Sigma-Aldrich Production GmbH, Switzerland) masses were typically ~5-10 mg and were measured on a Mettler balance with 0.001mg readability. 90-degree pulse excitation was used with 48 scans and a 1 minute recycle time. Data were processed using Mnova (Mestrelab Research) software.

N-(bicyclo[2.2.1]heptan-2-yl)-N′-(3,7-dimethylocta-2,6-dien-1-yl)ethane-1,2-diamine, ***10.***

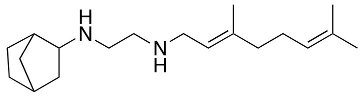

A mixture of the isoprenyl diamine (*E*)-*N′*-(3,7-dimethylocta-2,6-dienyl)ethane-1,2-diamine[1,2] (0.3 g, 2.72 mmol) and norcamphor (0.445 g, 2.27 mmol) in anhydrous methanol (15 mL) was stirred for 2 hours under nitrogen at room temperature. The reaction vessel was cooled to 0 ºC and the resulting imine was reduced by adding $NaBH_4$ (0.10 g, 2.27 mmol) gradually, the reaction was allowed to warm to room temperature and stirred overnight. The reaction mixture was concentration *in vacuo* and water (15 mL) was added to quench excess $NaBH_4$, the aqueous solution was extracted with ethyl acetate (3 x 20 mL), and the combined organic phase was washed with brine (20 mL), and the solution was dried over Na_2SO_4 and concentrated *in vacuo.* The obtained crude product was purified via column chromatography on silica gel using $CHCl_3$:CH_3OH:NH_4OH (88:10:2) as eluent to give a colorless oil (0.35 g, 53% yield) and converted to its HCl salt (an off-white solid; melting point: 165 ºC – 169 ºC) using a 1 M HCl solution in diethyl ether. Free base; ^{1}H NMR ($CDCl_3$, 300 MHz): δ_H = 5.26 (m, 1H), 5.08 (m, 1H), 3.26 (2H, d, J = 9.0 Hz), 3.04 – 2.97 (m, 1H), 2.76 – 2.61 (4H), 2.28 – 2.25 (m, 1H), 2.14 – 1.94 (m, 5H), 1.93 – 1.82 (m, 3H), 1.65 (s, 3H), 1.61 (s, 3H), 1.57 (s, 3H), 1.52 – 1.41 (m,1H), 1.36 – 1.12 (m, 5H), 0.68 – 0.57 (m, 1H). ^{13}C NMR ($CDCl_3$, 75 MHz); δ_C = 137.82, 131.56, 124.19, 122.79, 59.76, 49.28, 48.08, 47.15, 39.70, 39.50, 38.19, 38.15, 36.74, 30.25, 26.56, 25.77, 20.61, 17.75, 16.37; HRMS (TOF-ESI) calculated for $C_{19}H_{34}N_2$ ($[M + H]^+$) 291.2800; found 291.2799.

N-(bicyclo[2.2.2]octan-2-yl)-N′-(3,7-dimethylocta-2,6-dien-1-yl)ethane-1,2-diamine, ***11****.*

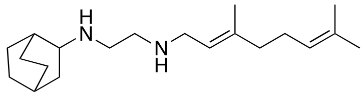

A mixture of isoprenyl diamine[1,2] (0.174 g, 0.885 mmol) and bicyclo[2,2,2]octan-2-one (0.10 g, 0.805 mmol) in anhydrous methanol (10 mL) was stirred for 2 hours under nitrogen at room temperature. The reaction vessel was cooled to 0 ºC using an ice-bath and the resulting imine was reduced by adding $NaBH_4$ (0.046 g, 1.21 mmol) gradually, the reaction was allowed to warm to room temperature and stirred overnight. The reaction mixture was concentration in vacuo and water (15 mL) was added to quench excess $NaBH_4$, the aqueous solution was extracted with ethyl acetate (3 x 20 mL), and the combined organic phase was washed with brine (20 mL), and the solution was dried over Na_2SO_4 and concentrated in vacuo. The crude product was purified via column chromatography on silica gel using $CHCl_3$:CH_3OH:NH_4OH (88:10:2) as eluent to give an off-white semi-solid (0.13 g, 53% yield) and converted to its HCl salt (an off-white solid; melting point: 161 ºC – 165 ºC) using 1M HCl in diethyl ether. Free base; ^{1}H NMR ($CDCl_3$, 300 MHz): δ_H = 5.28 (m, 1H), 5.07 (m, 1H), 3.93 (s, 3H), 3.36 (d, 2H, J = 6.0 Hz), 2.93 – 2.86 (m, 4H), 2.10 – 1.80 (m, 6H), 1.70 – 1.24 (m, 18H). ^{13}C NMR ($CDCl_3$, 75 MHz); δ_C = 140.47, 131.88, 123.98, 120.22, 56.12, 46.97, 46.25, 45.28, 39.77, 34.63, 27.77, 26.54, 25.83, 25.63, 25.13, 24.90, 24.53, 19.51, 17.83, 16.55; HRMS (TOF-ESI) calculated for $C_{20}H_{36}N_2$ ($[M + H]^+$) 305.2957; found 305.2964

N-benzyl-N'-(3,7-dimethylocta-2,6-dien-1-yl)ethane-1,2-diamine, ***12***.

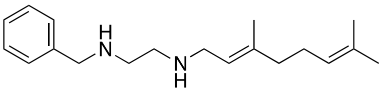

To a solution of isoprenyl diamine[1,2] (0.46 g, 2.34 mmol) in anhydrous methanol (15 mL) was added benzaldehyde (0.298 g, 2.81 mmol) and stirred for 2 hours under nitrogen at room temperature. The reaction vessel was cooled to 0 °C and the resulting imine was reduced by adding $NaBH_4$ (0.11 g, 2.81 mmol) gradually, the reaction was allowed to warm to room temperature and stirred overnight. The reaction mixture was concentration *in vacuo* and water (15 mL) was added to quench excess $NaBH_4$, the aqueous solution was extracted with ethyl acetate (3 x 20 mL), and the combined organic phrase was washed with brine (20 mL), and the solution was dried over Na_2SO_4 after which it was concentrated *in vacuo*. The crude product was purified via column chromatography on silica gel using $CHCl_3$:CH_3OH:NH_4OH (88:10:2) as eluent to give a white semi-solid (0.41 g, 61% yield) and converted to its HCl salt (a off-white solid; melting point: 258 °C – 261 °C) using 1M HCl in diethyl ether. Free base; ^{1}H NMR ($CDCl_3$, 300 MHz): δ_H = 7.32 – 7.23 (m, 5H), 5.24 (m, 1H), 5.09 (m, 1H), 3.79 (s, 2H), 3.22 (d, 2H, *J* = 6.0 Hz), 2.78 – 2.71 (m, 4H), 2.09 – 2.00 (m, 6H), 1.67 (s, 3H), 1.62 (s, 3H), 1.59 (s, 3H). ^{13}C NMR ($CDCl_3$, 75 MHz); δ_C = 140.47, 137.95, 131.58, 128.44, 128.20, 126.97, 124.18, 122.68, 54.02, 48.83, 47.10, 39.70, 26.57, 25.77, 17.76, 16.37; HRMS (TOF-ESI) calculated for $C_{19}H_{30}N_2$ ($[M + H]^+$) 287.2487; found 287.2502.

N-(adamantan-2-yl)-N'-(3,7-dimethylocta-2,6-dien-1-yl)propane-1,3-diamine, ***13***.

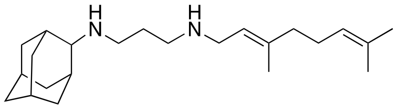

In a 1000 mL round-bottom flask was placed 1,3-propanediamine (20 mL) and dichloromethane (250 mL, reagent grade) and the solution stirred at -78°C. To this solution was added dropwise a solution of crude geranyl bromide[1,2] (1.78 mL, 9.18 mmol) in dichloromethane (150 mL), the resulting solution was then stirred overnight at room temperature under nitrogen. The organic solution was washed with water (4 x 200 mL) to remove excess diamine, brine (100 mL) and then dried over Na_2SO_4, the organic solution was concentrated *in vacuo* to obtain a crude product which was purified via column chromatography on silica gel using $CHCl_3$:CH_3OH:NH_4OH (88:10:2) as eluent to yield *N*-geranyl propane-1,3-diamine as a yellowish oil (1.12g, 58%), used in the next step.

A mixture of the *N*-geranyl propane-1,3-diamine (0.37 g, 1.76 mmol) and 2-adamantanone (0.24 g, 1.60 mmol) in anhydrous methanol (10 mL) was stirred for 2 hours under nitrogen at room temperature. The reaction vessel was cooled to 0 °C and the resulting imine was reduced by adding $NaBH_4$ (0.067g, 1.76 mmol) gradually, the reaction was allowed to warm to room temperature and stirred overnight. The reaction mixture was concentration *in vacuo* and water (30 mL) was added to quench the reaction, the aqueous solution was extracted with ethyl acetate (3 x 20mL), and the combined organic phrase was washed with brine (20mL), and the solution was dried over Na_2SO_4 and concentrated *in vacuo*. The crude product was purified via column chromatography on silica gel using $CHCl_3$:CH_3OH:NH_4OH (88:10:2) as eluent to give an off-white semi-solid (0.23 g, 42% yield) and converted to its HCl salt (a white solid; melting point: 276 °C – 279 °C) using 1M HCl in diethyl ether. Free base; ^{1}H NMR ($CDCl_3$, 300 MHz): δ_H = 5.25 (t, 1H, J = 6.0 Hz), 5.08 (t, 1H, J = 6.0 Hz), 3.23 (d, 2H, J = 9.0 Hz) 2.71 – 2.65 (m, 5H), 2.09 – 1.81 (m, 13H), 1.78 – 1.65 (m, 10H), 1.63 (s, 3H), 1.59 (s, 3H), 1.51 (d, 2H, J = 12.0 Hz). ^{13}C NMR ($CDCl_3$, 75 MHz); δ_C = 137.80, 131.61, 124.24, 122.84, 62.00, 48.66, 47.45, 45.94, 39.74, 38.05, 37.65, 32.01, 31.46, 30.50, 27.92, 27.72, 26.60, 25.81, 17.78, 16.40; HRMS (TOF-ESI) calculated for $C_{23}H_{41}N_2$ 9[M + H]) 345.3270; found 345.3268.

N-(adamantan-2-yl)-N'-(3,7-dimethylocta-2,6-dien-1-yl)-N'-methylethane-1,2-diamine, ***14***.

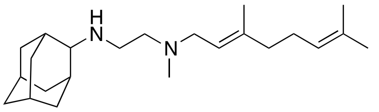

To a solution of SQ109 (0.5 g, 1.51 mmol) in CH_3OH (10 mL) was added 37% formaldehyde in water (0.71 mL, 9.06 mmol) and stirred at room temperature for 1 hour then heated at 50°C for an additional hour under nitrogen. The solution was then cooled using an ice-bath before $NaBH_4$ (0.37 g, 9.82 mmol) was added slowly and the resulting solution was stirred at room temperature overnight. The reaction mixture was concentrated *in vacuo*, the crude residue was dissolved

in ethyl acetate (30 mL) and washed successively with water (2 x 20 mL) and brine (20 mL). The organic phase was dried over Na_2SO_4 and concentrated *in vacuo* and the crude product was purified via column (silica gel) chromatography using $CHCl_3$:CH_3OH:NH_4OH (88:10:2) as eluent to afford **14** as a white semi-solid; yield 0.15 g (29%) and converted to its HCl salt (a white solid; melting point: 191 °C – 195 °C) using 1M HCl in diethyl ether. Free base; ^{1}H NMR ($CDCl_3$, 300 MHz): δ_H = 5.23 (m, 1H), 5.09 (m, 1H), 3.00 (d, 2H, J = 9.0 Hz), 2.72 (m, 3H), 2.52 (t, 2H, J = 6.0 Hz), 2.20 (s. 3H), 2.13 – 1.78 (m, 13H), 1.75 – 1.65 (m, 7H), 1.63 (s, 3H), 1.60 (s, 3H), 1.52 (d, 2H, J = 12.0 Hz). ^{13}C NMR ($CDCl_3$, 75 MHz); δ_C = 138.56, 131.62, 124.29, 121.51, 62.05, 57.04, 55.39, 44.51, 42.13, 39.93, 38.05, 37.73, 32.02, 31.44, 27.92, 27.68, 26.61, 25.84, 17.81, 16.50; HRMS (TOF-ESI) calculated for $C_{23}H_{41}N_2$ ([M + H]$^+$) 345.3270; found 345.3279.

N-(adamantan-2-yl)-N′-phenethylethane-1,2-diamine, ***22****.*

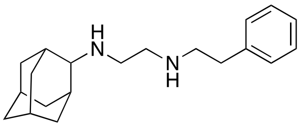

To a stirred solution of ethylene diamine (1.5 g, 25 mmol) in 10 mL of anhydrous methanol was added slowly phenylacetaldehyde (1.39 mL, 12.5 mmol) over 5 minutes, the solution was stirred at room temperature for 2 hours after which it was cooled to 0 °C before slowly adding $NaBH_4$ (0.57 g, 14.98 mmol), the resulting solution was stirred overnight at room temperature. The reaction mixture was concentrated *in vacuo* and water (20 mL) was added to quench excess $NaBH_4$ after which the solution was extracted with ethyl acetate (3 x 20 mL) and washed with brine (20 mL). The organic phase was dried using Na_2SO_4 and concentrated *in vacuo* to afford the crude product which was purified via column (silica gel) chromatography using $CHCl_3$:CH_3OH:NH_4OH (88:10:2) as eluent to afford *N*-phenethylethane-1,2-diamine (1.17 g, 57% yield) which was then used in the next step.

In a 50 mL round bottom flask was placed *N*-phenethylethane-1,2-diamine (0.65 g, 3.96 mmol), 2-adamantanone (0.66 g, 4.39 mmol) and anhydrous methanol (15 mL), the resulting solution was then stirred at room temperature for 2 hours. The solution was then cooled using an ice-bath before $NaBH_4$ (0.166 g, 4.36 mmol) was added slowly and the resulting solution was stirred at room temperature overnight. The reaction mixture was concentrated *in vacuo*, water (20 mL) was added to the crude product and extracted with ethyl acetate (2 x 20mL), the combined organic layer was then washed with brine (20 mL). The organic phase was dried over Na_2SO_4 and concentrated *in vacuo* and the crude product was purified via column (silica gel) chromatography using $CHCl_3$:CH_3OH:NH_4OH (88:10:2) as eluent to afford **22** as a white semi-solid (0.68 g, 57 % yield) and converted to its HCl salt (an off-white solid; melting point: 286 °C – 291 °C) using 1M HCl in diethyl ether. Free base; ^{1}H NMR ($CDCl_3$, 300 MHz): δ_H = 7.31 – 7.26 (m, 2H), 7.23 – 7.17 (m, 3H), 2.92 – 2.87 (m, 2H), 2.83 – 2.78 (m, 2H), 2.76 – 2.66 (m, 5H), 1.96 – 1.78 (m, 7H), 1.77 – 1.63 (m, 5H), 1.47 (d, 2H, *J* = 15.0 Hz), 1.30 (s, 2H). ^{13}C NMR ($CDCl_3$, 75 MHz); δ_C = 140.31, 128.82, 128.49, 126.14, 61.90, 51.21, 50.00, 46.51, 38.07, 37.67, 36.61, 32.27, 31.38, 27.92, 27.73; HRMS (TOF-ESI) calculated for $C_{20}H_{30}N_2$ ([M + H]$^+$) 299.2487, found 299.2493.

N-(adamantan-2-yl)-N′-(3-phenoxybenzyl)ethane-1,2-diamine, ***23****.*

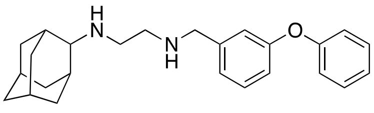

To a stirred solution of ethylene diamine (0.91 g, 15.1 mmol) in 10 mL of anhydrous methanol was added slowly 3-phenoxybenzaldehyde (1.5g, 7.57 mmol) over 5 minutes, the solution was stirred at room temperature for 2 hours after which it was cooled to 0 °C before slowly adding $NaBH_4$ (0.34 g, 9.08 mmol), the resulting solution was stirred overnight at room temperature. The reaction mixture was concentrated *in vacuo* and water (20 mL) was added to quench excess $NaBH_4$ after which the solution was extracted with ethyl acetate (3 x 20 mL) and washed with brine (20 mL). The organic phase was dried using Na_2SO_4 and concentrated *in vacuo* to afford the crude product which was purified via column (silica gel) chromatography using $CHCl_3$:CH_3OH:NH_4OH (88:10:2) as eluent to afford *N*-(3-phenoxybenzyl)ethane-1,2-diamine (1.09 g, 59% yield) as a colorless oil which was used in the next step.

In a 50 mL round bottom flask was placed *N*-(3-phenoxybenzyl)ethane-1,2-diamine (0.65 g, 2.68 mmol), 2-adamantanone (0.443 g, 2.95 mmol) and anhydrous methanol (15 mL), the resulting solution was then stirred at room temperature for 2 hours. The solution was then cooled to 0 °C before $NaBH_4$ (0.134 g, 3.54 mmol) was added slowly and the resulting solution was stirred at room temperature overnight. The reaction mixture was concentrated *in vacuo*, water (20 mL) was added to the crude product and extracted with ethyl acetate (2 x 20 mL), the combined organic layer was then washed with brine (20 mL). The organic phase was dried over Na_2SO_4 and concentrated *in vacuo* and the crude product was purified via column (silica gel) chromatography using $CHCl_3$:CH_3OH:NH_4OH (88:10:2) as eluent to afford **23** as a colorless oil, (0.61 g, 59 % yield) and converted to its HCl salt (a white solid; melting point: 266 °C – 269 °C)

using 1M HCl in diethyl ether. Free base; ^{1}H NMR ($CDCl_3$, 300 MHz): δ_H = 7.37 – 7.25 (m, 3H), 7.12 – 6.99, (m, 5H), 6.91 – 6.87 (m, 1H), 3.78 (s, 2H), 2.78 – 2.68 (m, 5H), 1.96 (d, 2H, J = 12.0 Hz), 1.86 – 1.62 (m, 12H), 1.50 (d, 2H, *J* = 12.0 Hz). ^{13}C NMR ($CDCl_3$, 75 MHz); δ_C = 157.45, 157.35, 142.86, 129.82, 129.73, 123.27, 123.04, 118.97, 118.58, 117.40, 61.95, 53.66, 49.25, 46.49, 38.04, 37.68, 32.17, 31.44, 27.89, 27.69; HRMS (TOF-ESI) calculated for $C_{25}H_{32}N_2O$ ([M + H]$^+$) 377.2593; found 377.2596.

N-(adamantan-2-yl)-N'-(2-cyclohexylethyl)ethane-1,2-diamine, ***24.***

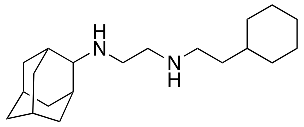

To solution of 2-cyclohexylethanol (2 g, 15.6 mmol) in anhydrous dichloromethane (60 mL) was added pyridinium chlorochromate (5 g, 23.2 mmol), silica gel (5 g) and celite (5 g) and the mixture stirred overnight under nitrogen. The resulting reaction mixture was filtered via silica gel and celite until the filtrate becomes colorless. The filtrate was concentrated in vacuo to obtain the corresponding crude aldehyde (2-cyclohexylacetaldehyde) as a yellow oil (1.3 g) and used in the next step without further purification.

To a stirred solution of ethylenediamine (1.24 g, 20.6 mmol) in 20 mL of anhydrous methanol was added slowly 2-cyclohexylacetaldehyde (1.3 g, 10.3 mmol) over 5 minutes, the solution was stirred at room temperature for 2 hours after which it was cooled to 0 ^{o}C before slowly adding $NaBH_4$ (0.47 g, 12.4 mmol), the resulting solution was stirred overnight at room temperature. The reaction mixture was concentrated *in vacuo* and water (20 mL) was added to quench excess $NaBH_4$ after which the solution was extracted with ethyl acetate (3 x 20 mL) and washed with brine (20 mL). The organic phase was dried using Na_2SO_4 and concentrated *in vacuo* to afford the crude product which was purified via column (silica gel) chromatography using $CHCl_3$:CH_3OH:NH_4OH (88:10:2) as eluent to afford *N*-(2-cyclohexylethyl)ethane-1,2-diamine (0.61 g, 35% yield) as a colorless oil which was used in the next step.

In a 50 mL round bottom flask was placed *N*-(2-cyclohexylethyl)ethane-1,2-diamine (0.60 g, 3.5 mmol), 2-adamantanone (0.635 g, 4.23 mmol) and anhydrous methanol (20 mL), the resulting solution was then stirred at room temperature for 2 hours. The solution was then cooled to 0 ^{o}C before $NaBH_4$ (0.2 g, 5.3 mmol) was added slowly and the resulting solution was stirred at room temperature overnight. The reaction mixture was concentrated *in vacuo*, water (30 mL) was added to the crude product and extracted with ethyl acetate (3 x 20 mL), the combined organic layer was then washed with brine (20 mL). The organic phase was dried over Na_2SO_4 and concentrated *in vacuo* and the crude product was purified via column (silica gel) chromatography using $CHCl_3$:CH_3OH:NH_4OH (88:10:2) as eluent to afford **24** as a colorless oil (0.3686 g, 35% yield) and converted to its HCl salt (a white solid; melting point: 284 ^{o}C – 287 ^{o}C) using 1M HCl solution in diethyl ether. Free base ^{1}H NMR ($CDCl_3$, 300 MHz): δ_H = 2.69 – 2.66 (m, 5H), 2.59 (t, 2H, J = 6.0, 9.0 Hz), 1.94 – 1.89 (m, 4H), 1.86 – 1.76 (m. 5H), 1.75 – 1.53 (m, 10H), 1.50 – 1.08 (m, 8H), 0.96 – 0.79 (m, 2H). ^{13}C NMR ($CDCl_3$, 75 MHz); δ_C = 61.94, 50.00, 47.48, 46.36, 38.00, 37.71, 37.64, 35.7, 33.48, 32.19, 31.39, 27.86, 27.68, 26.67, 26.38; HRMS (TOF-ESI) calculated for $C_{20}H_{36}N_2$ ([M + H]$^+$) 305.2957; found 305.2957.

N-(adamantan-2-yl)-N'-benzylethane-1,2-diamine, ***25****.*

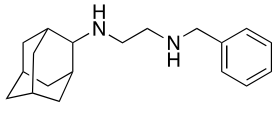

To a stirred solution of ethylene diamine (1.77 g, 29.5 mmol) in 20 mL of anhydrous methanol was added slowly benzaldehyde (1.57 g, 14.8 mmol) over 5 minutes, the solution was stirred at room temperature for 2 hours after which it was cooled to 0 ^{o}C before slowly adding $NaBH_4$ (1.12 g, 29.5 mmol), the resulting solution was stirred overnight at room temperature. The reaction mixture was concentrated *in vacuo* and water (40 mL) was added to quench excess $NaBH_4$ after which the solution was extracted with ethyl acetate (3 x 20 mL) and the combined organic layer was washed with brine (20 mL). The organic phase was dried using Na_2SO_4 and concentrated *in vacuo* to afford the crude product which was purified via column (silica gel) chromatography using $CHCl_3$:CH_3OH:NH_4OH (88:10:2) as eluent to afford *N*-benzylethane-1,2-diamine (1.0583 g, 48% yield) as a yellowish oil which was used in the next step.

In a 50 mL round bottom flask was placed *N*-benzylethane-1,2-diamine (1.06 g, 7.05 mmol), 2-adamantanone (0.988 g, 6.4 mmol) and anhydrous methanol (20 mL), the resulting solution was then stirred at room temperature for 2 hours. The solution was then cooled to 0 ^{o}C before $NaBH_4$ (0.484 g, 12.8 mmol) was added slowly and the resulting solution was stirred at room temperature overnight. The reaction mixture was concentrated *in vacuo*, water (30 mL) was added to the crude product and extracted with ethyl acetate (3 x 20 mL), the combined organic layer was then washed with brine (20 mL). The organic phase was dried over Na_2SO_4 and concentrated *in vacuo* and the crude product was purified

via column (silica gel) chromatography using $CHCl_3$:CH_3OH:NH_4OH (88:10:2) as eluent to afford **25** as a yellowish oil (0.69 g, 38% yield) and converted to its HCl salt (as a white solid; melting point: 293 °C – 296 °C) using 1M HCl solution in diethyl ether. Free base; ^{1}H NMR ($CDCl_3$, 300 MHz): δ_H = 7.29 – 7.22 (m, 4H), 7.21 – 7.16 (m, 1H), 3.75 (s, 2H), 2.69 – 2.60 (m, 5H), 1.92 (d, 2H, J = 12.0 Hz), 1.83 – 1.60 (m, 10H), 1.53 (s, 2H), 1.44 (d, 2H, J = 12.0 Hz). ^{13}C NMR ($CDCl_3$, 75 MHz); δ_C = 140.59, 128.30, 128.08, 126.78, 61.80, 53.88, 49.36, 46.46, 37.95, 37.57, 32.16, 31.33, 27.81, 27.62; HRMS (TOF-ESI) calculated for $C_{19}H_{28}N_2$ ([M + H]$^+$) 285.2331; found 285.2340.

N-(adamantan-2-yl)-N′-(3-methylbut-2-en-1-yl)ethane-1,2-diamine, ***26.***

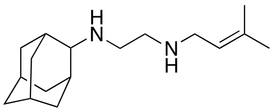

A solution of ethylene diamine (10 mL) in dichloromethane (100 mL) was stirred at -78°C and 3,3-dimethylallyl bromide in dichloromethane (150 mL) was added dropwise over 30 – 45 minutes, the resulting solution was then stirred overnight at room temperature under nitrogen. The organic solution was washed with water (3 x 100 mL) to remove excess diamine, brine (100 mL) and then dried over Na_2SO_4, the organic solution was concentrated *in vacuo* to obtain a crude product which was purified via column chromatography on silica gel using $CHCl_3$:CH_3OH:NH_4OH (88:10:2) as eluent to yield *N*-(3-methylbut-2-en-1-yl)ethane-1,2-diamine as a colorless oil (0.1941 g, 15%) which was used in the next step.

A mixture of the *N*-(3-methylbut-2-en-1-yl)ethane-1,2-diamine (0.194 g, 1.51 mmol) and 2-adamantanone (0.207 g, 1.38 mmol) in anhydrous methanol (10 mL) was stirred for 2 hours under nitrogen at room temperature. The reaction vessel was cooled to 0 °C and the resulting imine was reduced by adding $NaBH_4$ (0.104 g, 2.75 mmol) gradually, the reaction was allowed to warm to room temperature and stirred overnight. The reaction mixture was concentration in vacuo and water (20 mL) was added to quench the reaction, the aqueous solution was extracted with ethyl acetate (3 x 20 mL), and the combined organic phase was washed with brine (20 mL), and the solution was dried over Na_2SO_4 and concentrated *in vacuo*. The crude product was purified via column chromatography on silica gel using $CHCl_3$:CH_3OH:NH_4OH (88:10:2) as eluent to give an off-white semi-solid (0.27 g, 73% yield) and converted to its HCl salt (a off-white solid; melting point: 205 °C – 210 °C) using 1M HCl solution in diethyl ether. ^{1}H NMR ($CDCl_3$, 300 MHz): δ_H = 5.26 (t, 1H, J = 6.0 Hz), 3.24 (d, 2H, J = 9.0 Hz), 2.75 – 2.71 (m, 5H), 2.19 (s, 2H), 1.97 (d, 2H, J = 12.0 Hz), 1.86 – 1.75 (m, 6H), 1.74 – 1.66 (m, 7H), 1.64 (s, 3H), 1.51 (d, 2H, J = 12.0 Hz). ^{13}C NMR ($CDCl_3$, 75 MHz); δ_C = 134.80, 122.70, 62.06, 49.24, 47.09, 46.35, 38.03, 37.68, 32.16, 31.43, 27.89, 27.69, 25.89, 18.06. HRMS (TOF-ESI) calculated for $C_{17}H_{30}N_2$ ([M + H]$^+$) 263.2487; found 263.2495.

SUPPORTING INFORMATION FIGURES

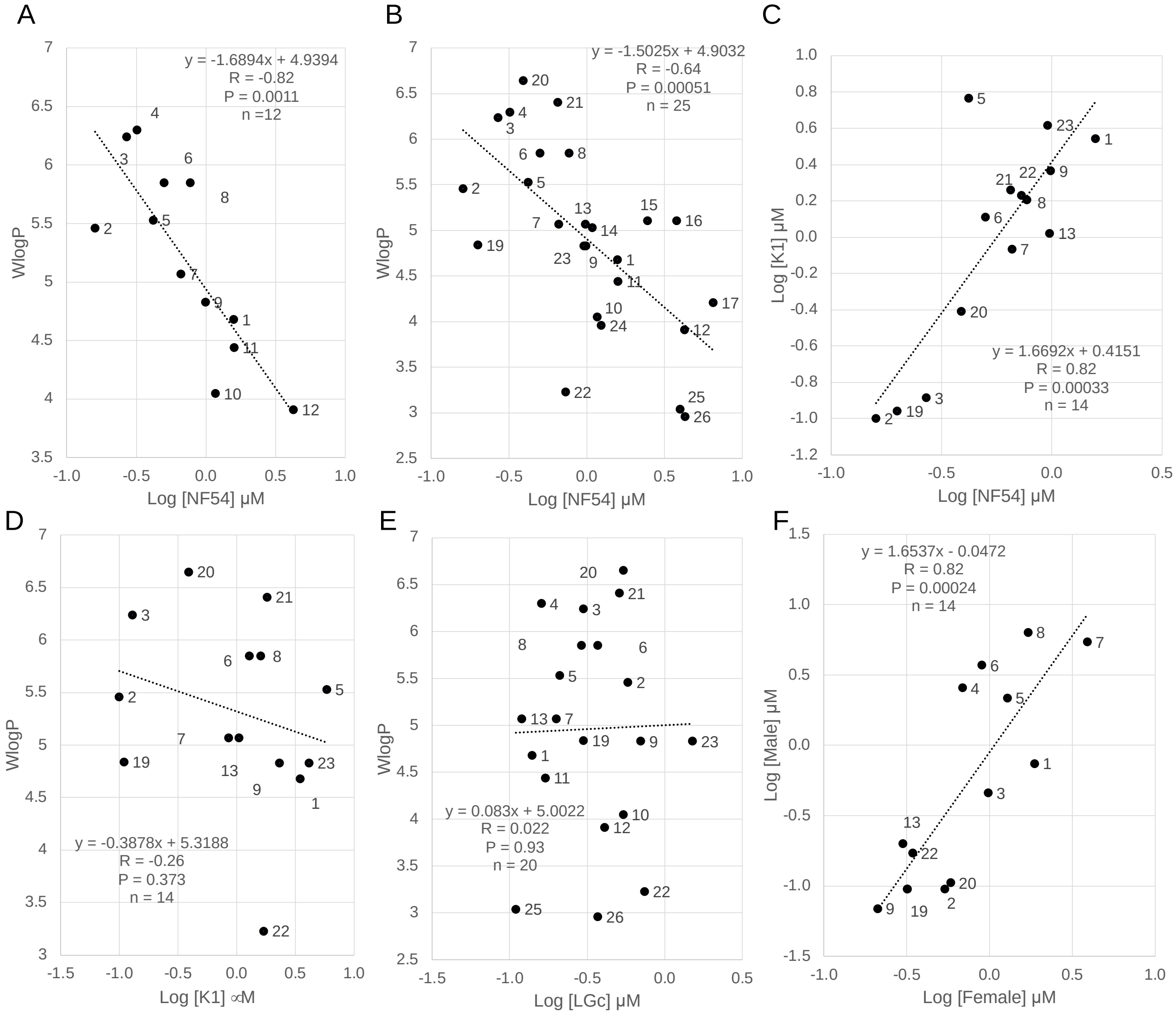

Figure S1. Correlations between *Pf*ABS and late-stage gametocyte viability inhibition by SQ109 and its analogs (log IC_{50}, μM) with WlogP; drug-sensitive (*Pf*NF54) and drug-resistant (*Pf*K1) inhibition activity correlations; and male/female gamete inhibition correlation results. (A) Correlation between *Pf*NF54 activity and WlogP for the 12 compounds in SAR-1: R=-0.82, P=0.0011. (B) As in A but for the 25 (out of 26 total) compounds with measurable activity in SAR-1, SAR-2 and SAR-3: R=-0.64, P = 0.00051. (C) Correlation between log IC_{50} for *Pf*NF54 and *Pf*K1 cell lines: R=0.82, n=14, P=0.00033. (D) Correlation between log *Pf*K1 IC_{50} and WlogP: R=-0.26, n=14, P=0.37. (E) Correlation between gametocyte inhibition (log *Pf*LGc, μM) and WlogP: R=0.022, n=20, P=0.93. (F) Correlation between log IC_{50} values for male and female gamete inhibition: R=0.82, n=14, P=0.00024.

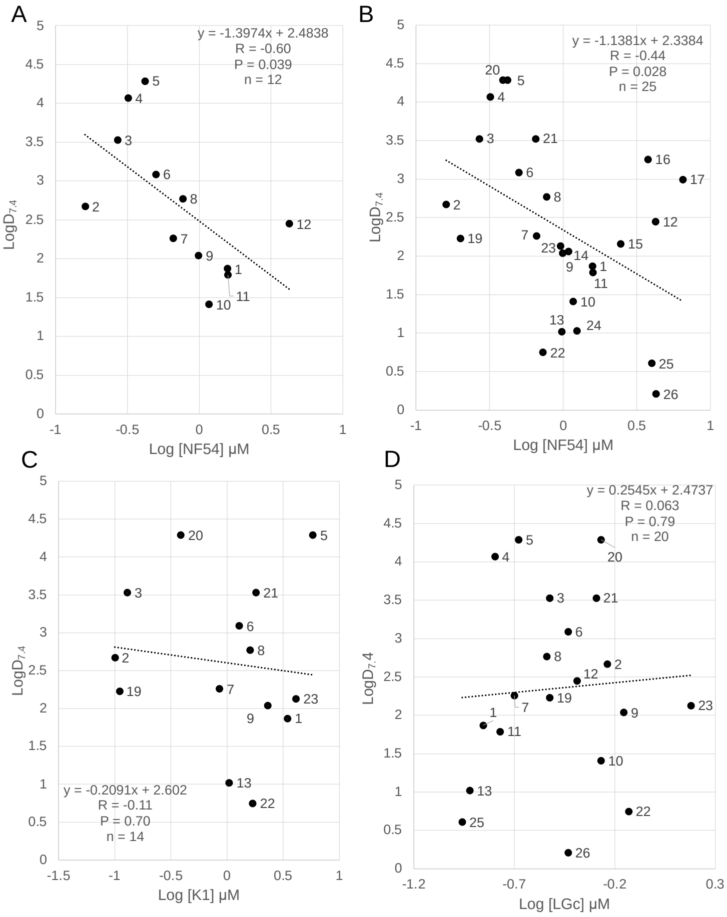

Figure S2. Correlations between *Pf*ABS and late-stage gametocyte viability inhibition by SQ109 and its analogs (log IC_{50}, µM) with $logD_{7.4.}$ (A) Correlation between *Pf*NF54 activity and $logD_{7.4}$ for the 12 compounds in SAR-1: R=-0.60, P=0.039. (B) As in A but for the 25 compounds with activity < 10 µM in SAR-1, SAR-2 and SAR-3: R=-0.44, P = 0.028. (C) Correlation between log *Pf*K1 IC_{50} and $logD_{7.4}$: R=-0.11, n=14, P=0.70. (D) Correlation between gametocyte inhibition (log *Pf*LGc, µM) and $logD_{7.4}$: R=0.63, n=20, P=0.79.

A

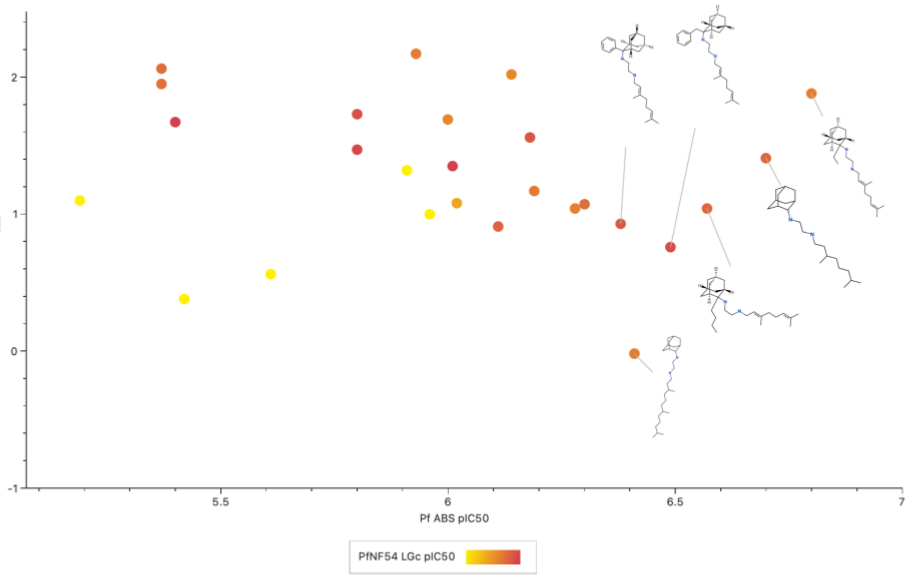

B

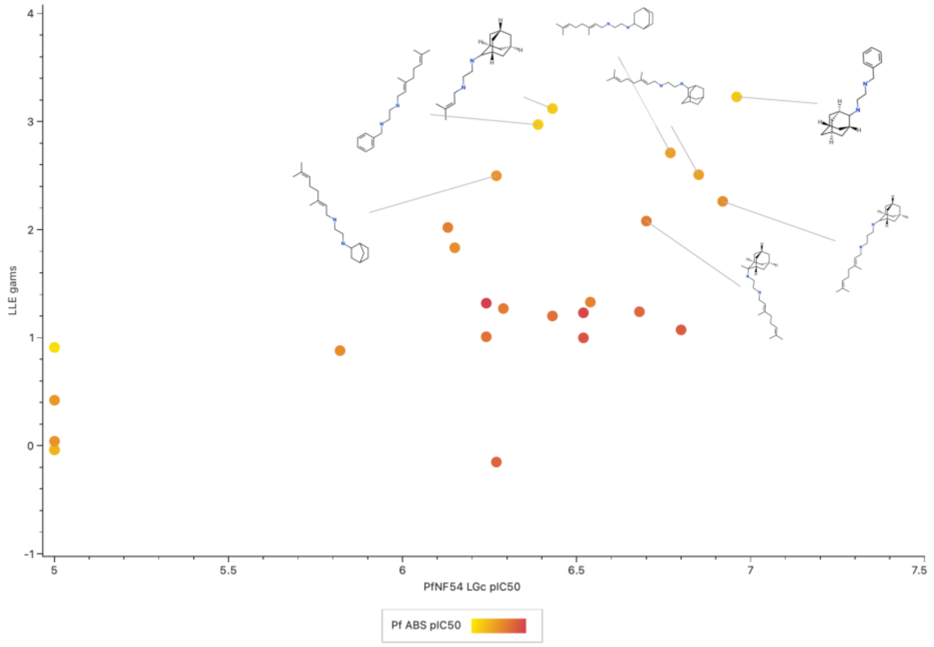

Figure S3. *Pf*ABS and late-stage gametocyte (LGc) activity by SQ109 and its analogs associated to ligand-lipophilicity efficiency (LLE). LLE was calculated for efficiency as (**A**) ABS activity (pIC_{50}^{ABS}-clogP) or efficiency against (**B**) late-stage gametocyte (pIC_{50}^{LG} – clogP). Calculations, clogP predictions and the plots were generated in StarDrop.

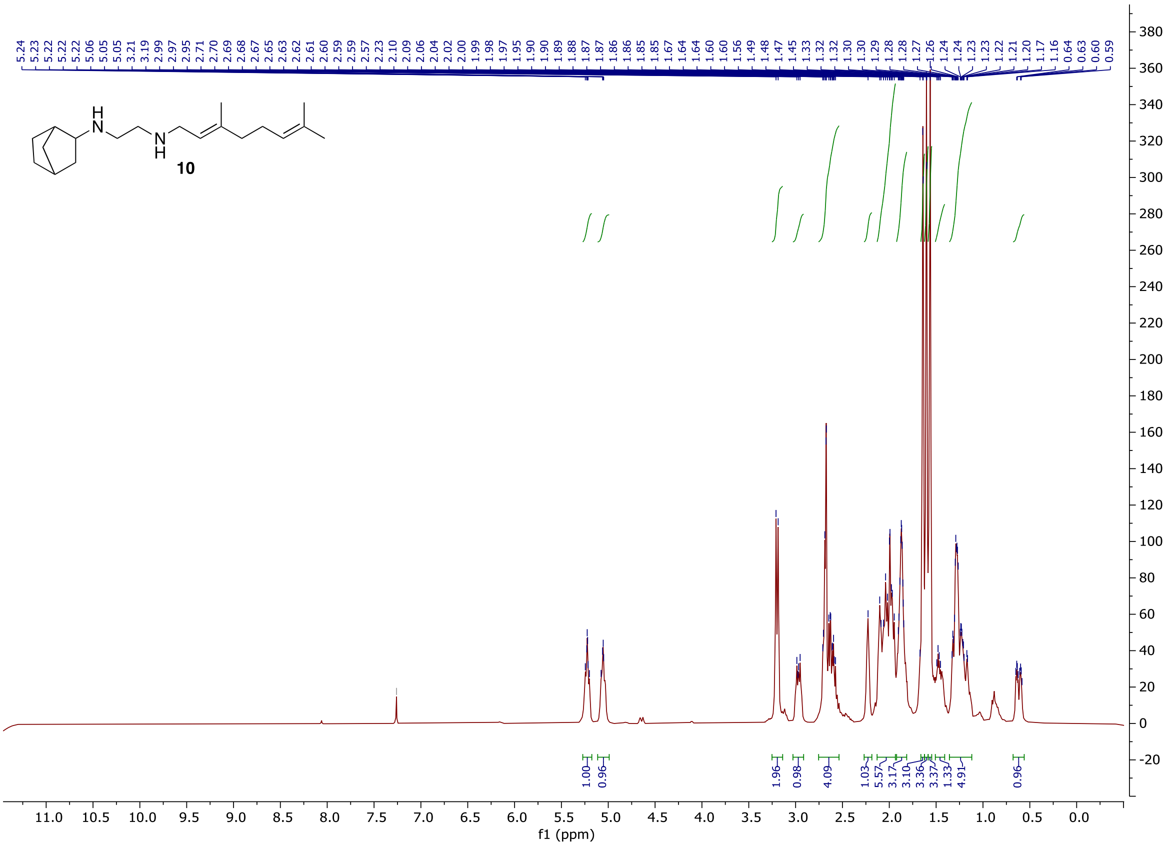

Figure S4: ^{1}H NMR spectrum of compound **10**

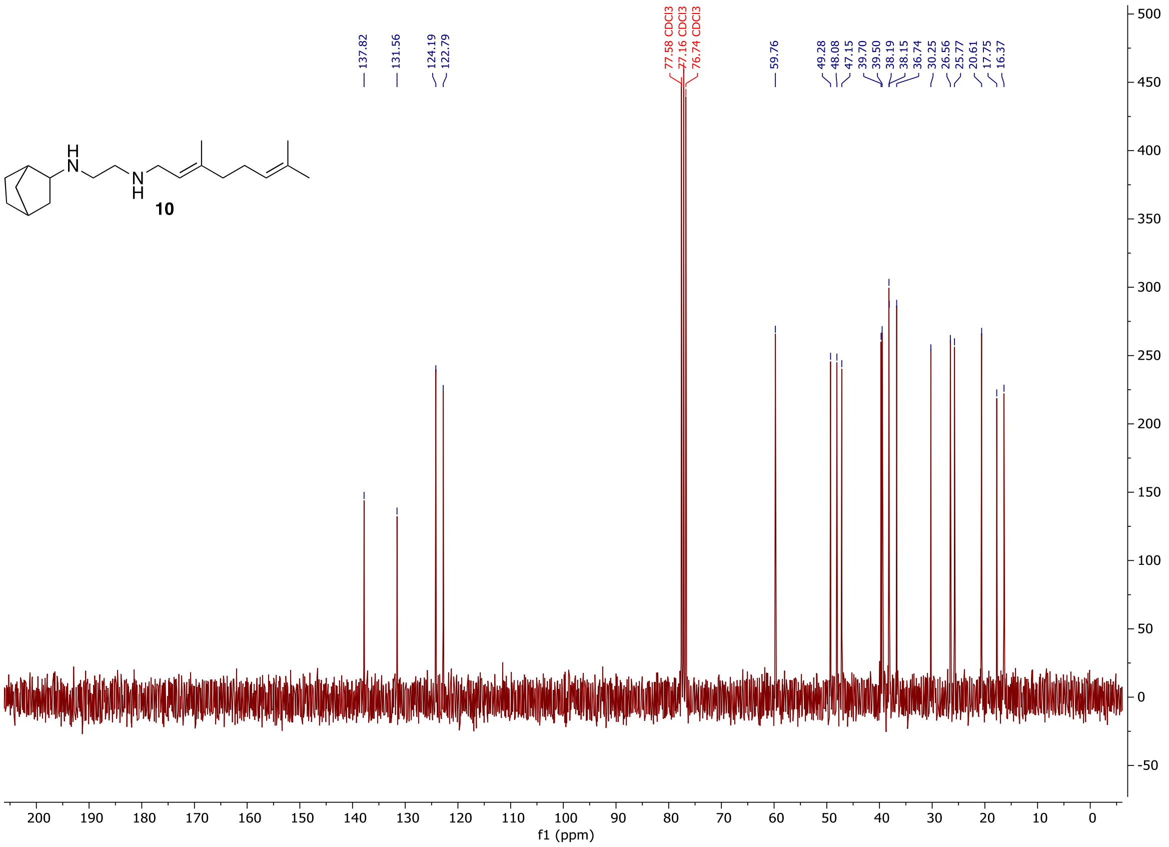

Figure S4: ^{13}C NMR spectrum of compound **10**

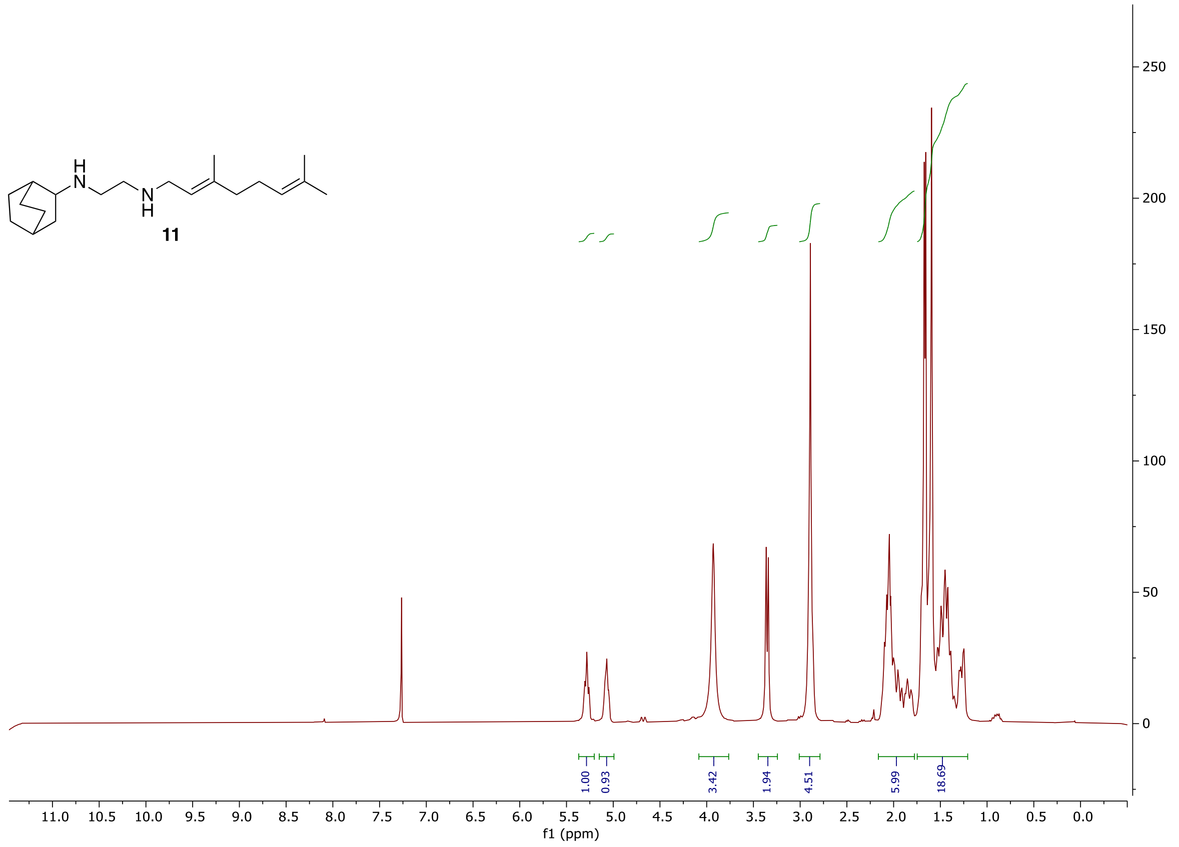

Figure S4: ^{1}H NMR spectrum of compound **11**

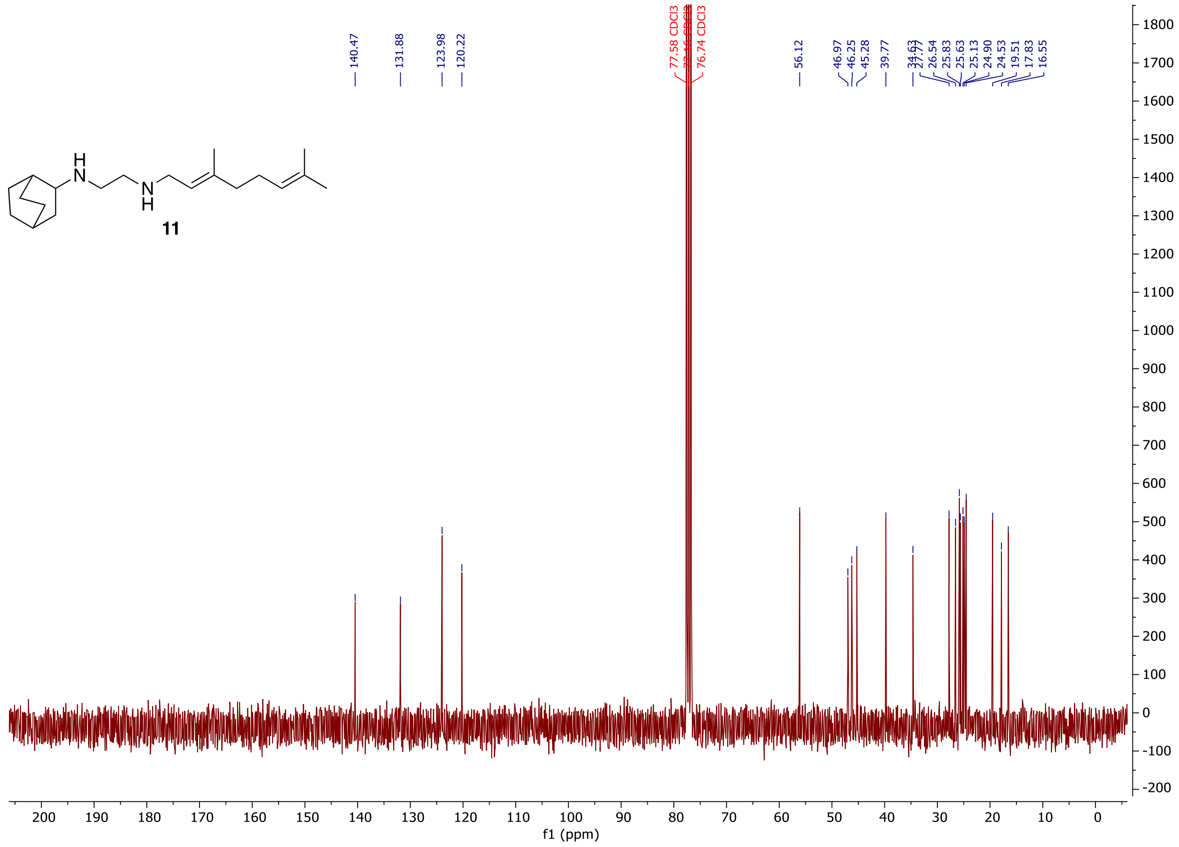

Figure S4: ^{13}C NMR spectrum of compound **11**

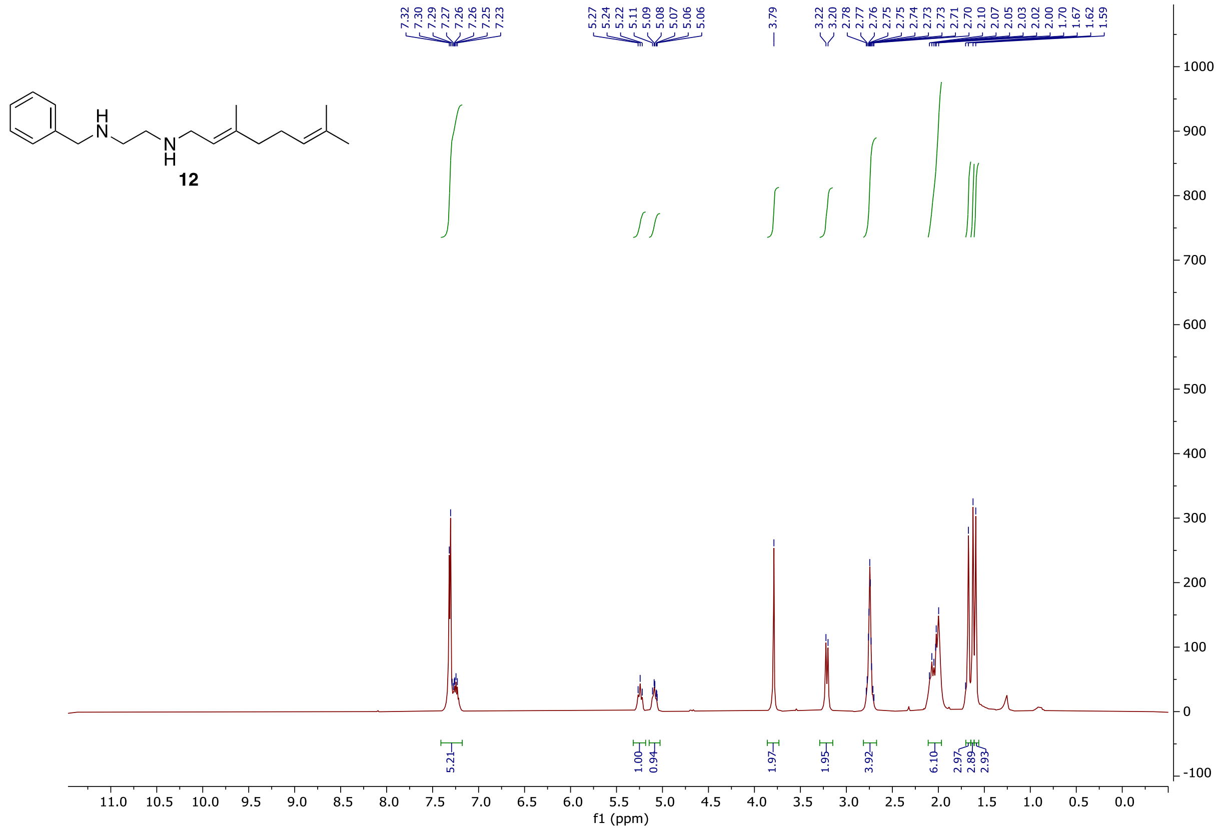

Figure S4: ^{1}H NMR spectrum of compound **12**

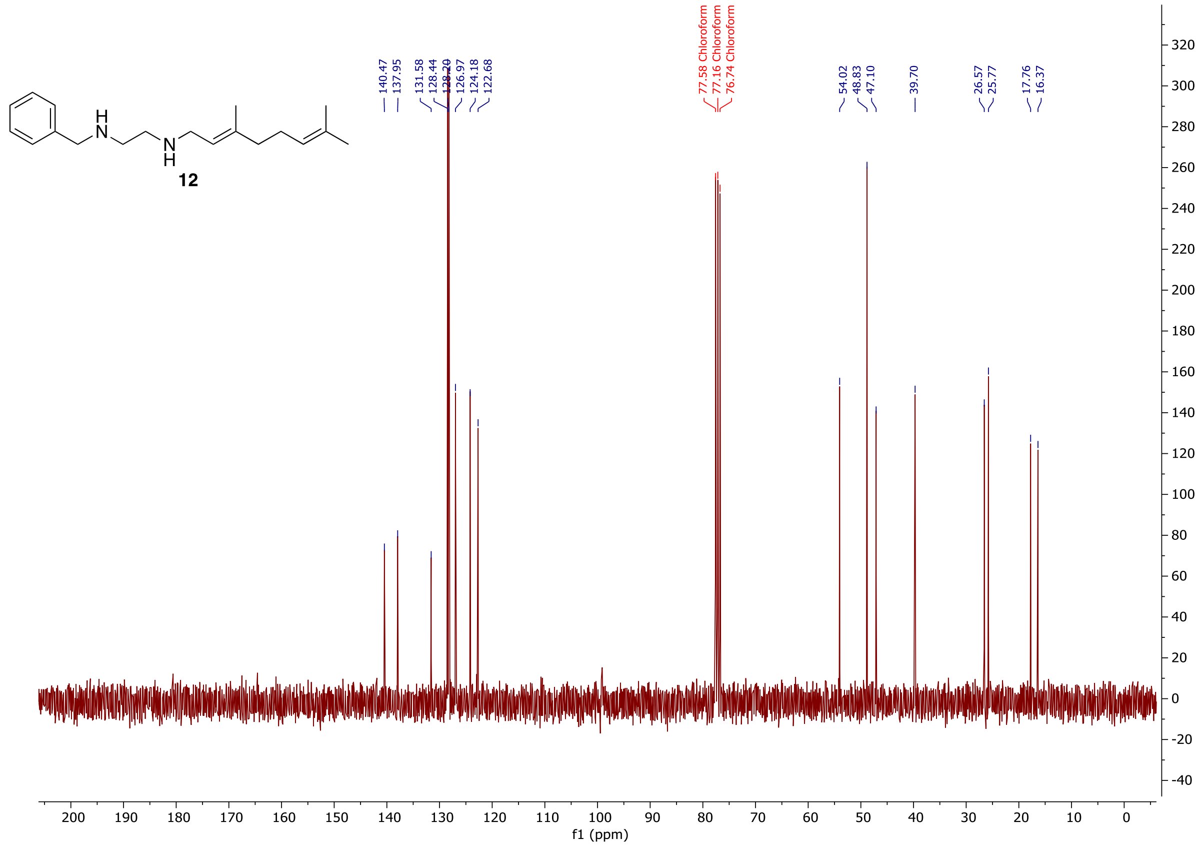

Figure S4: ^{13}C NMR spectrum of compound **12**

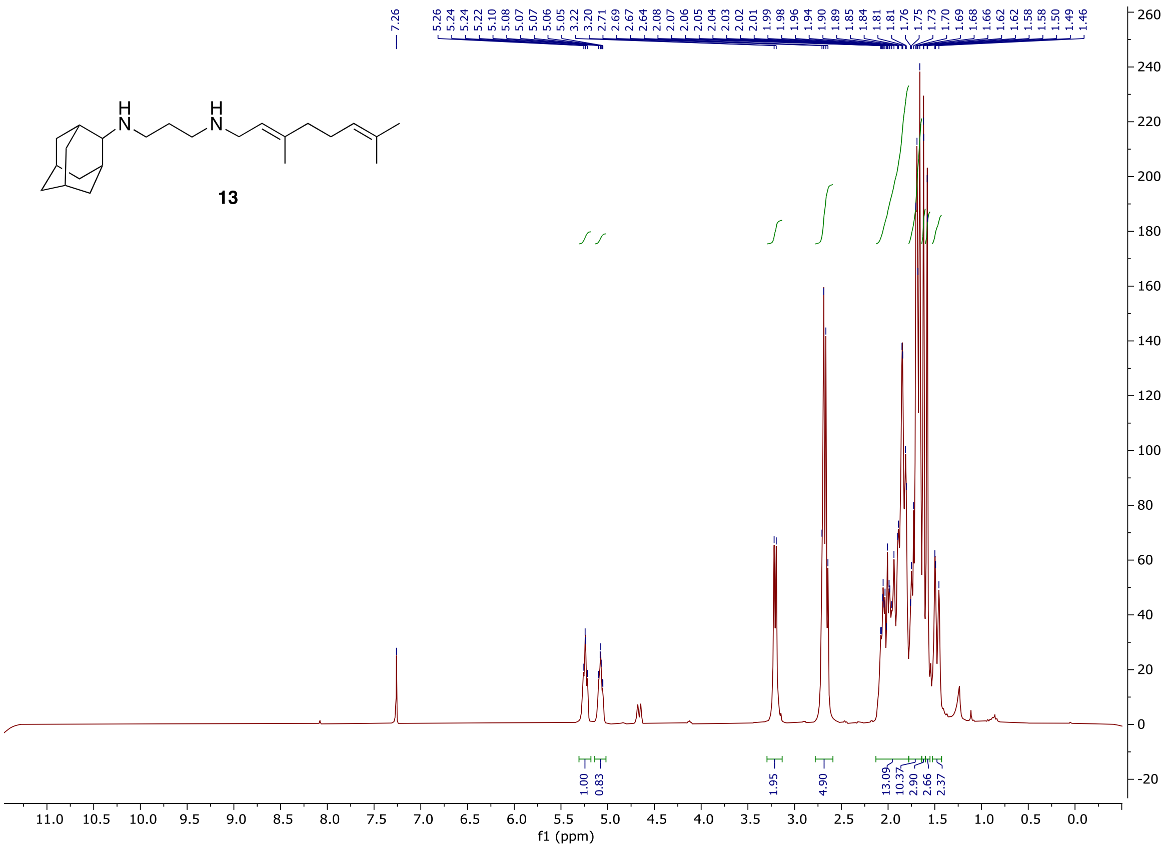

Figure S4: ^{1}H NMR spectrum of compound **13**

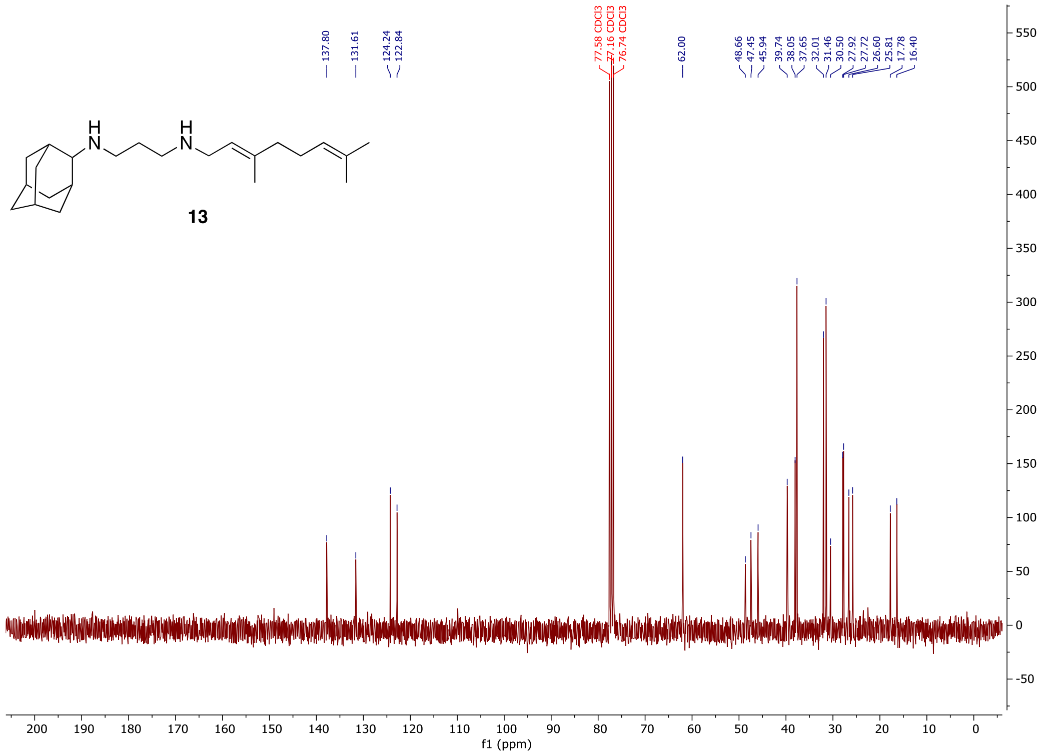

Figure S4: ^{13}C NMR spectrum of compound **13**

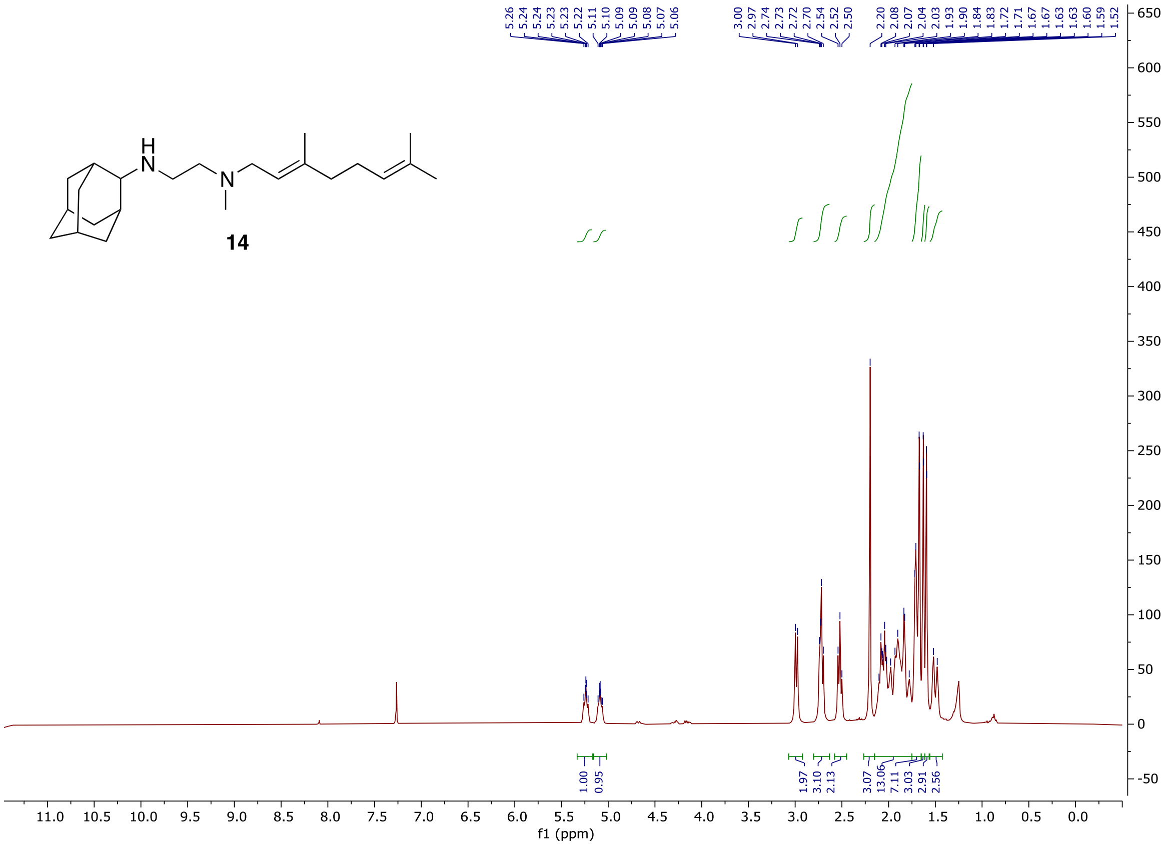

Figure S4: ^{1}H NMR spectrum of compound **14**

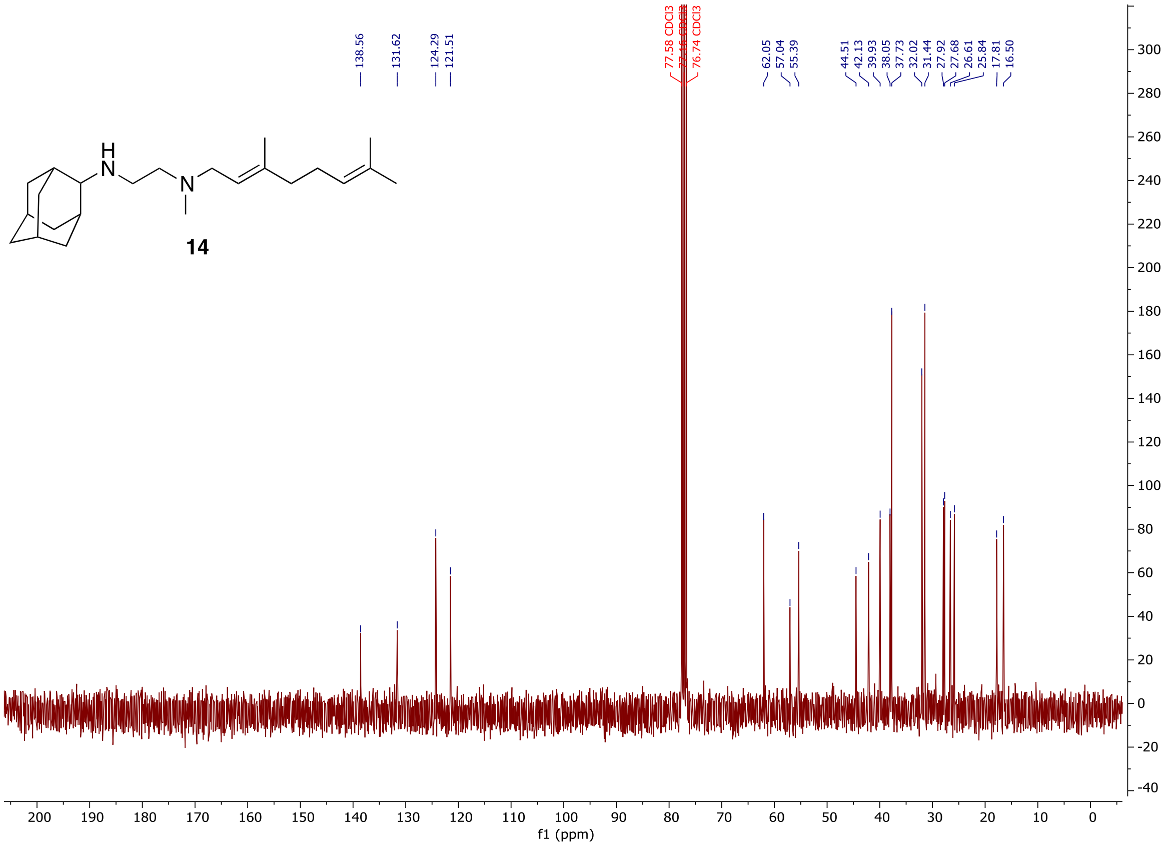

Figure S4: ^{13}C NMR spectrum of compound **14**

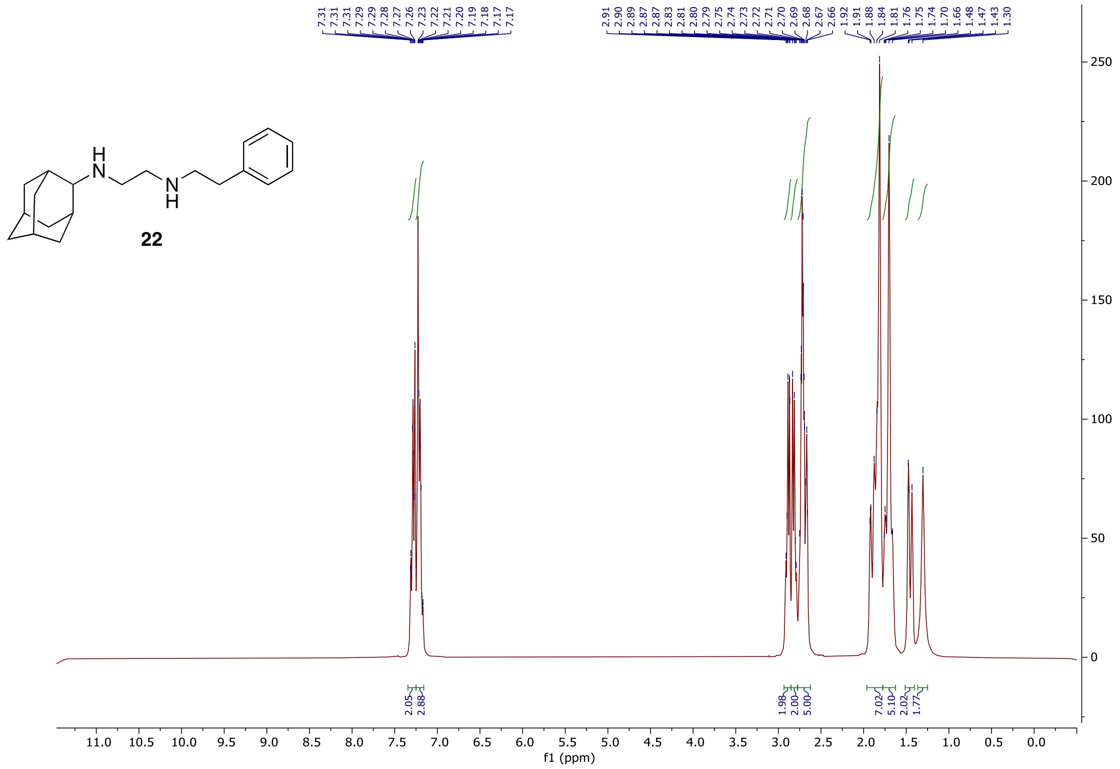

Figure S4: ^{1}H NMR spectrum of compound **22**

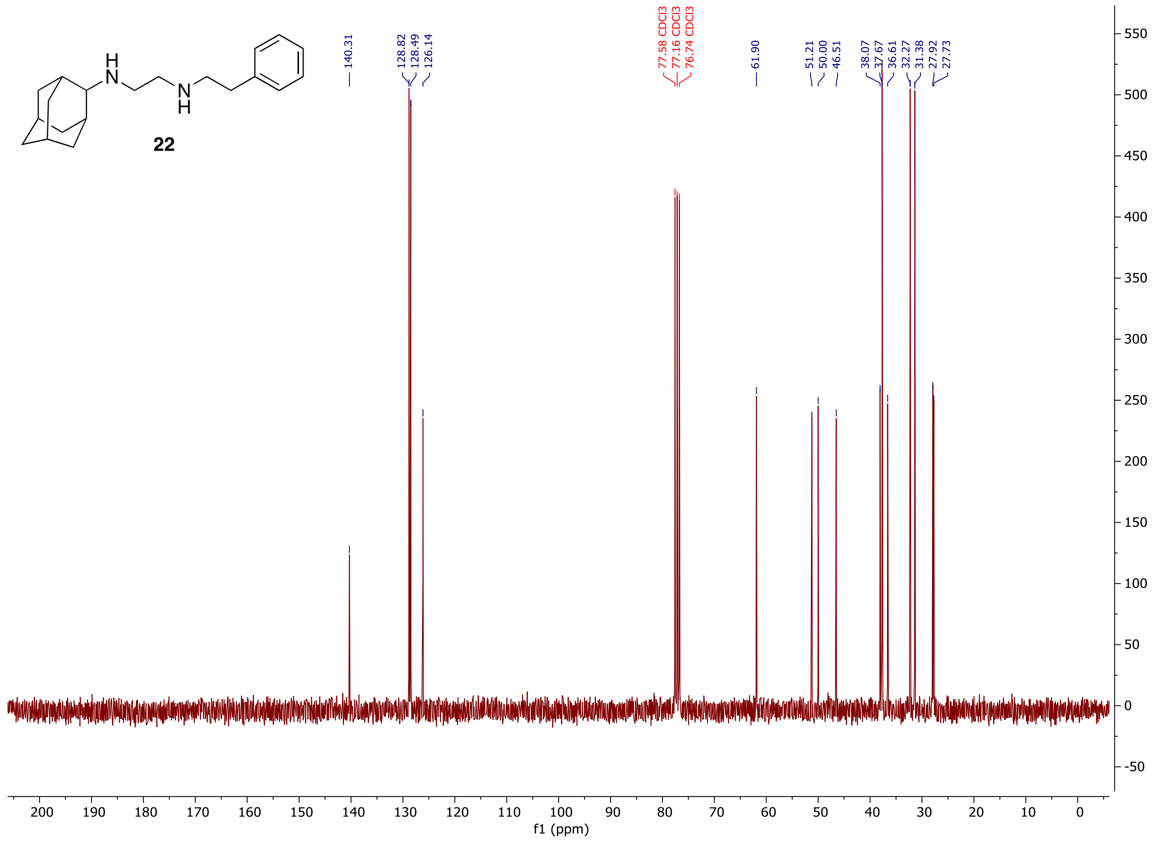

Figure S4: ^{13}C NMR spectrum of compound **22**

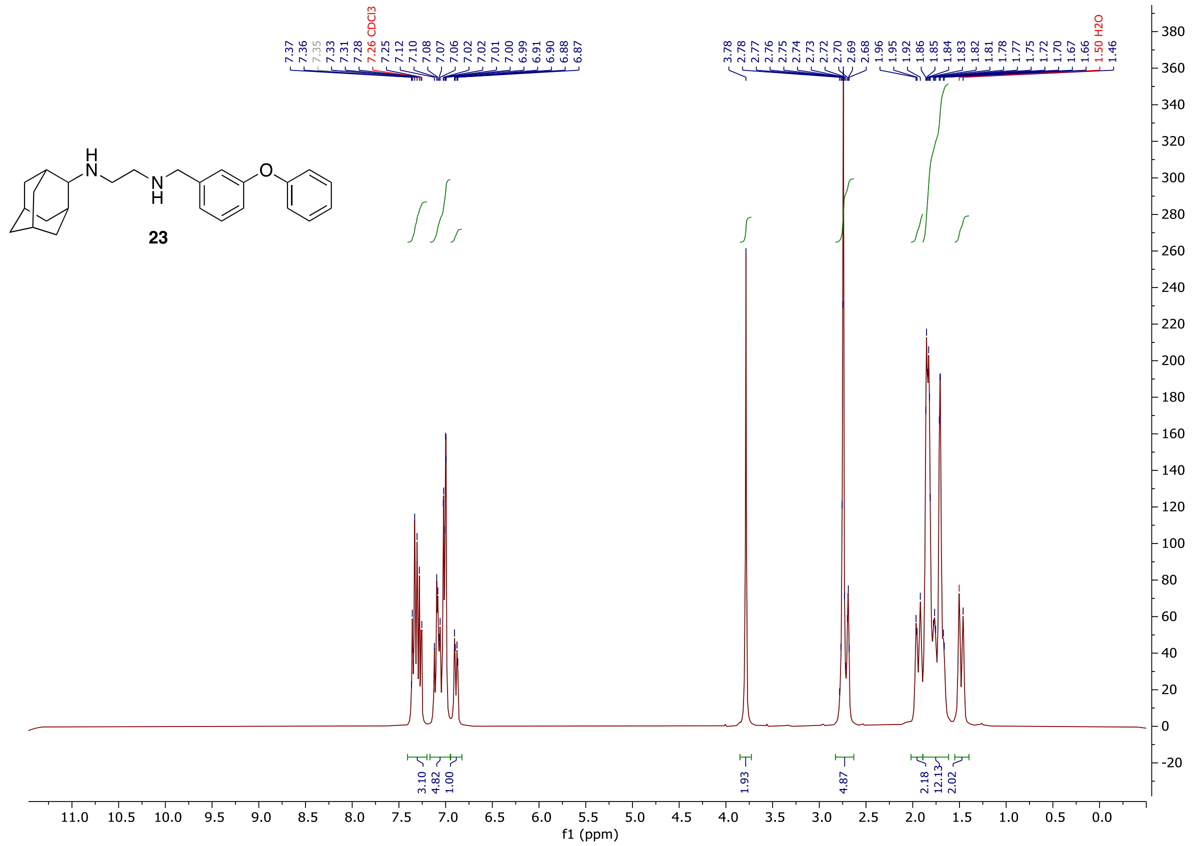

Figure S4: ^{1}H NMR spectrum of compound **23**

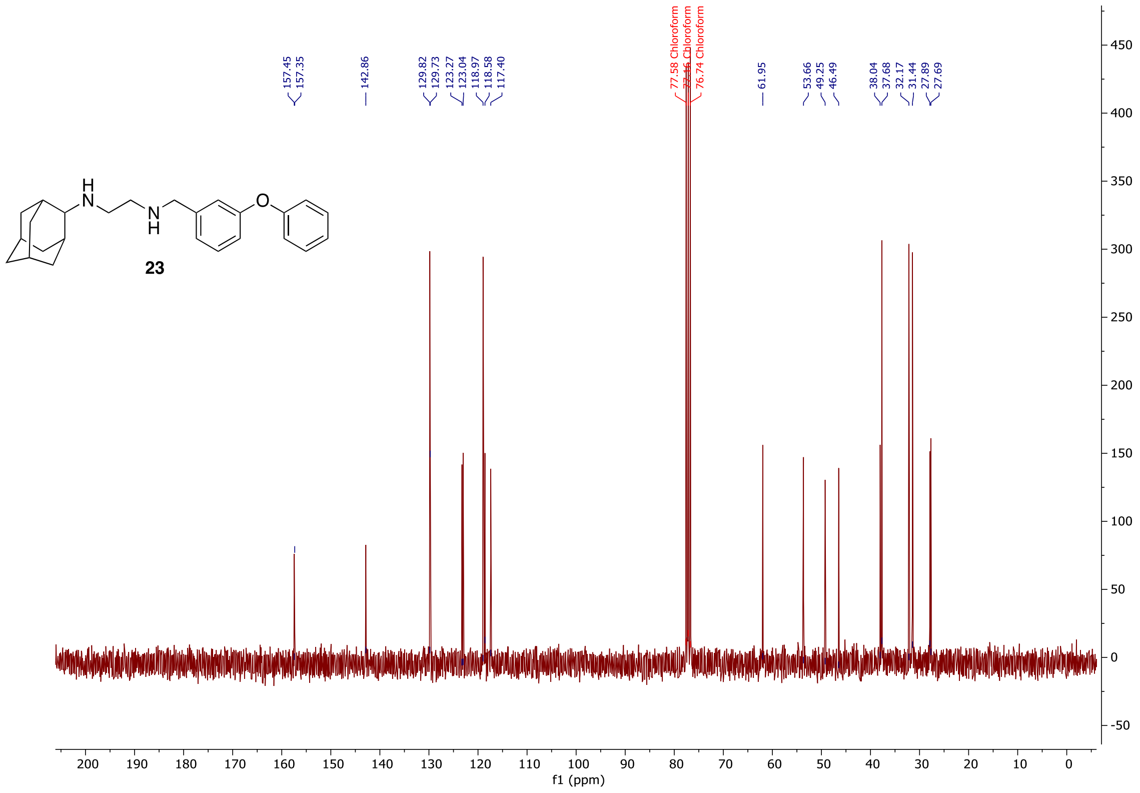

Figure S4: ^{13}C NMR spectrum of compound **23**

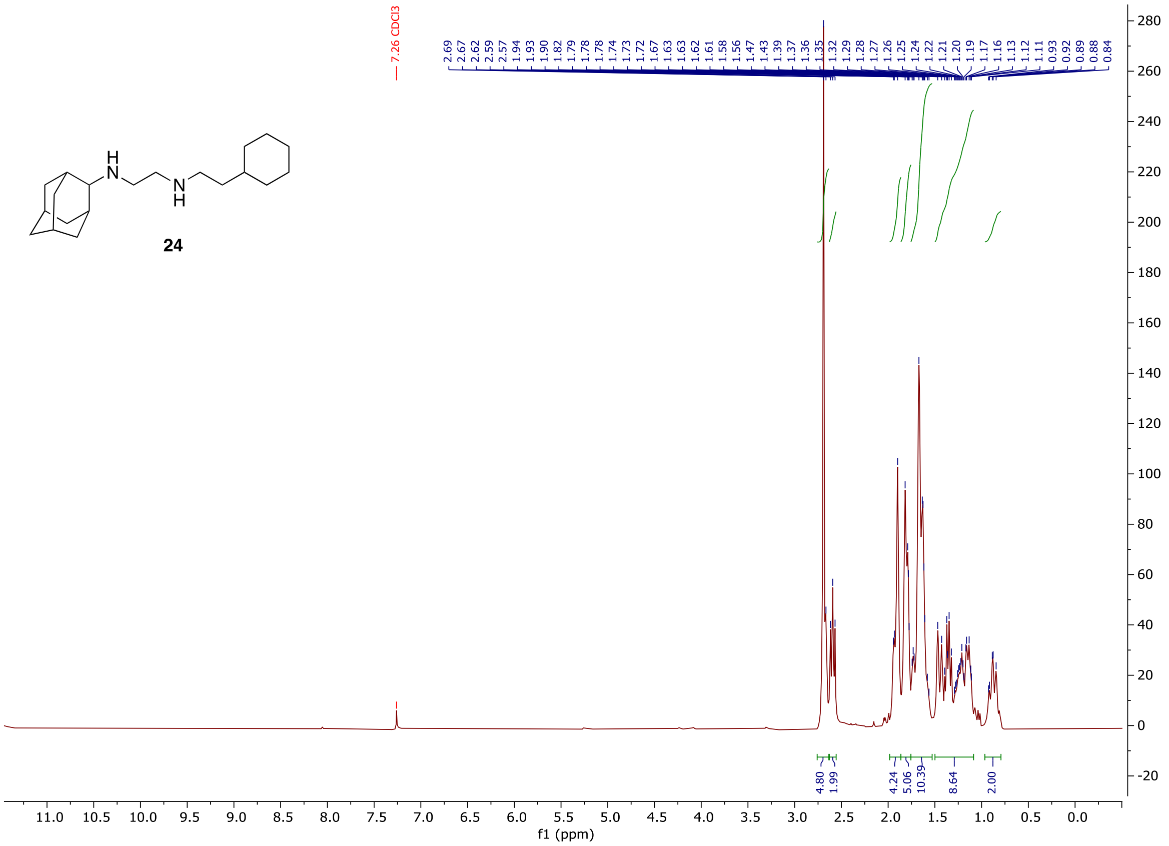

Figure S4: ^{1}H NMR spectrum of compound **24**

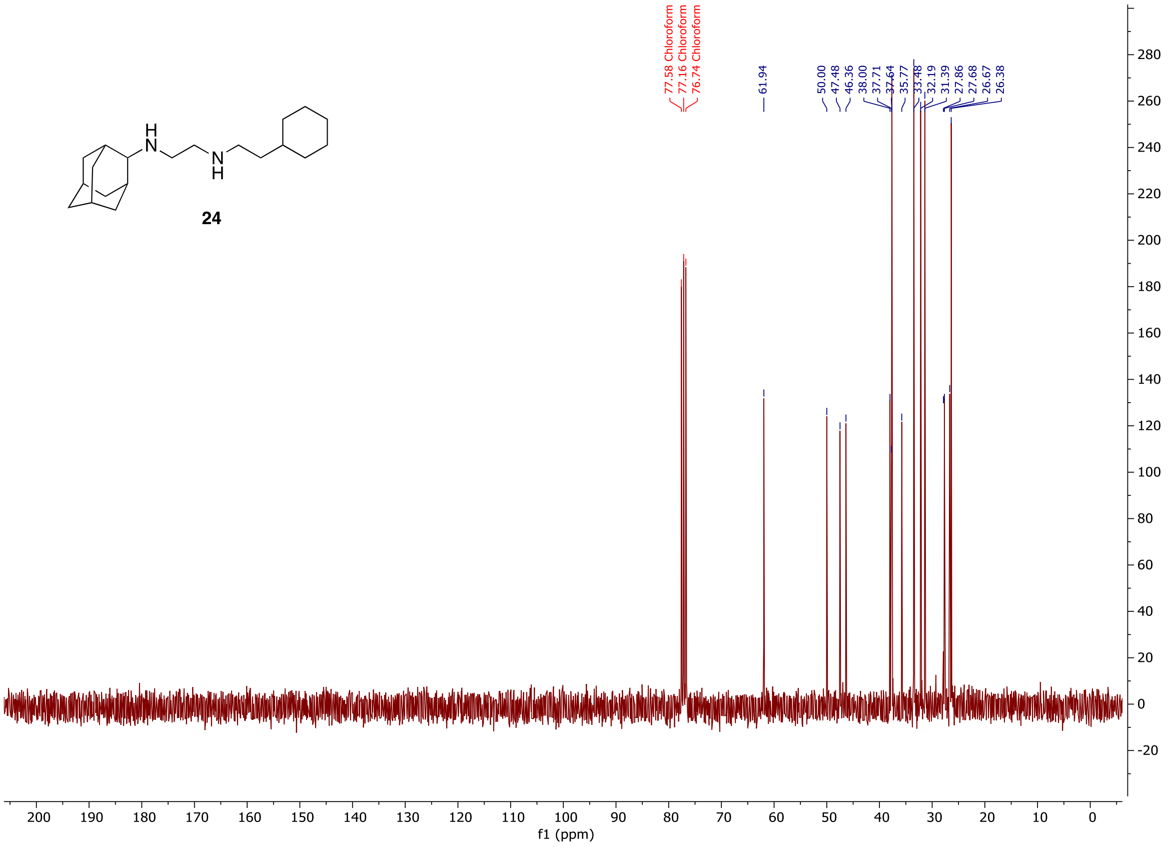

Figure S4: ^{13}C NMR spectrum of compound **24**

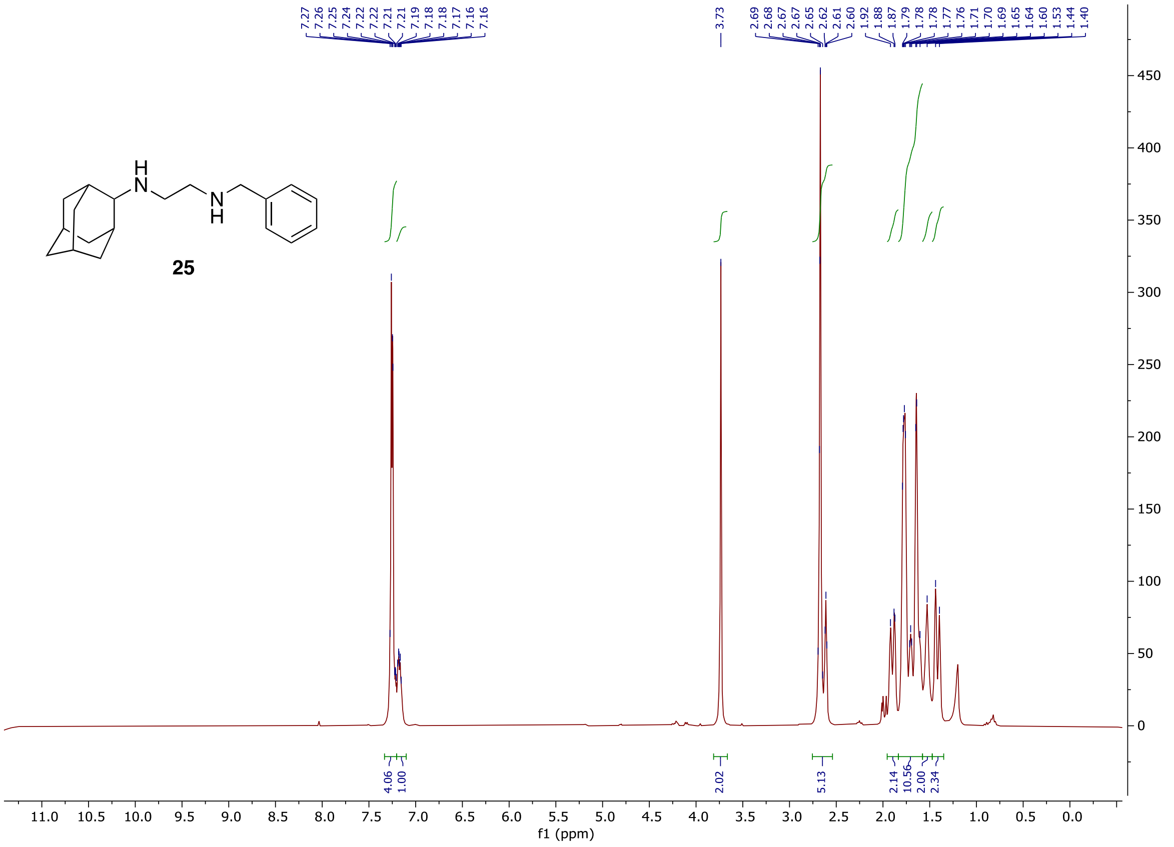

Figure S4: ^{1}H NMR spectrum of compound **25**

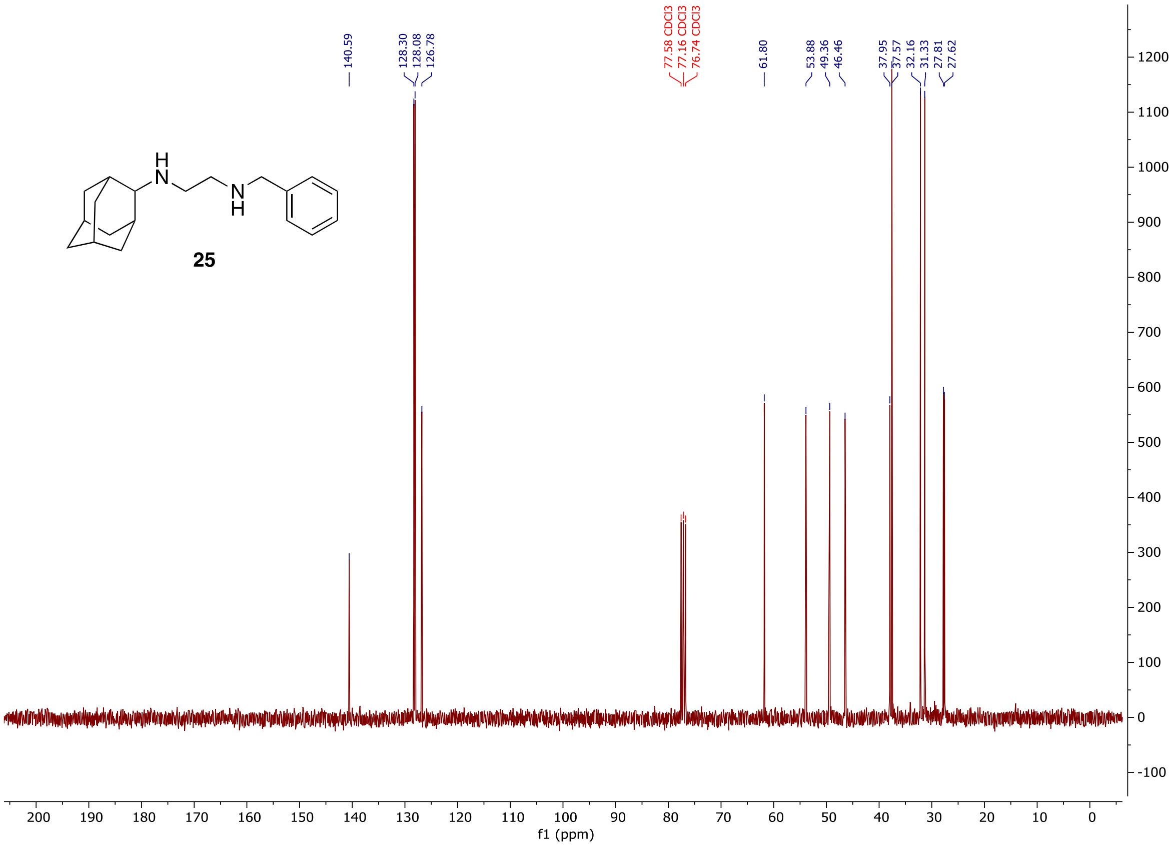

Figure S4: ^{13}C NMR spectrum of compound **25**

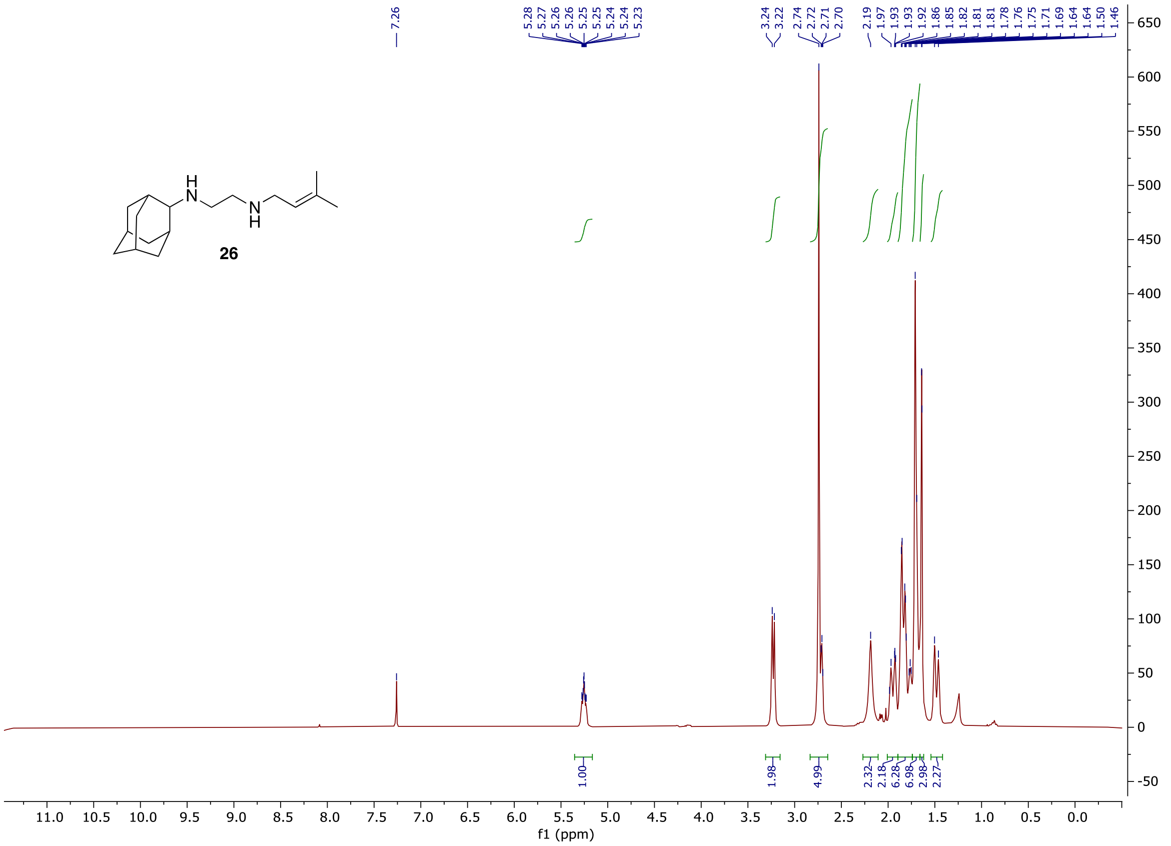

Figure S4: ^{1}H NMR spectrum of compound **26**

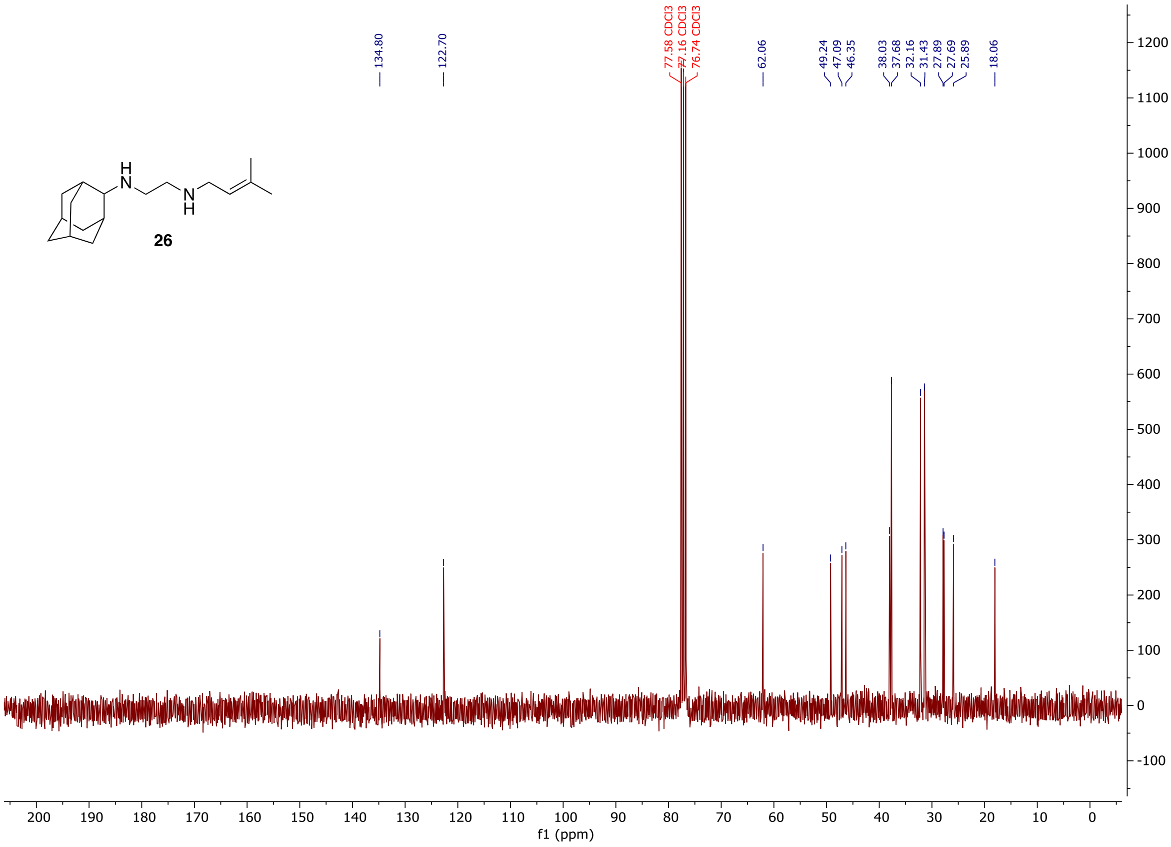

Figure S4: ^{13}C NMR spectrum of compound **26**

Figure S5. qNMR spectra and purity assessment:

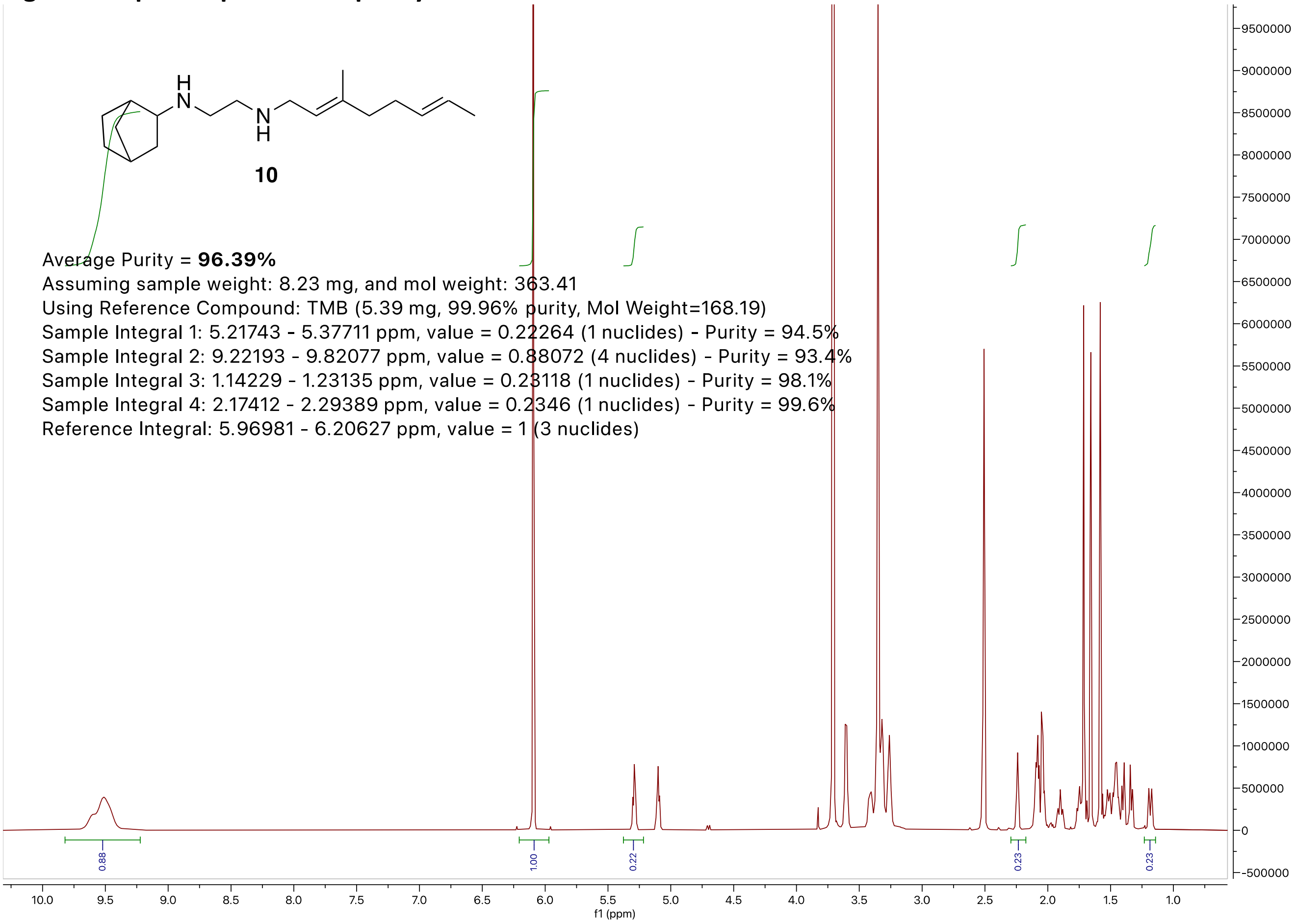

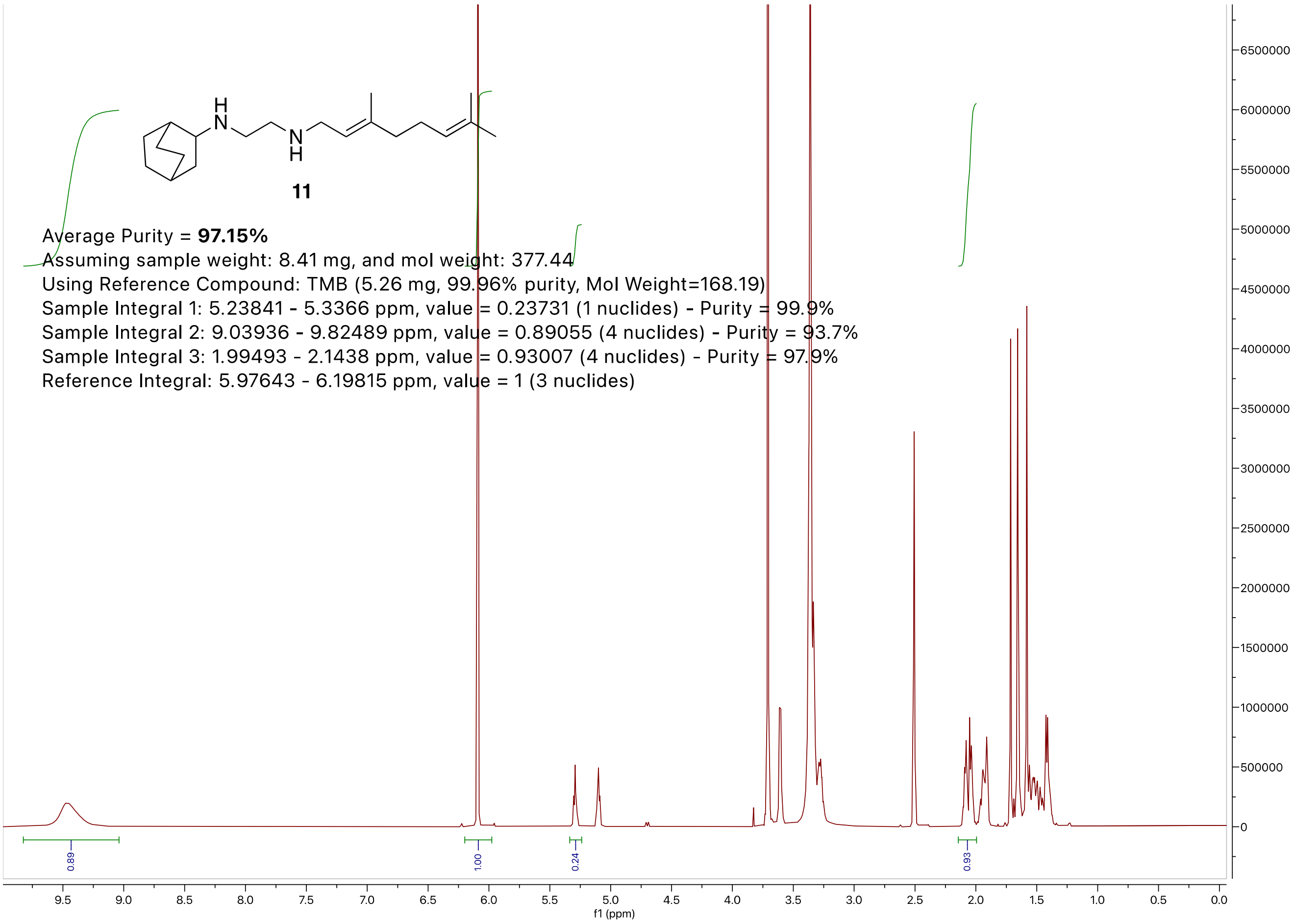

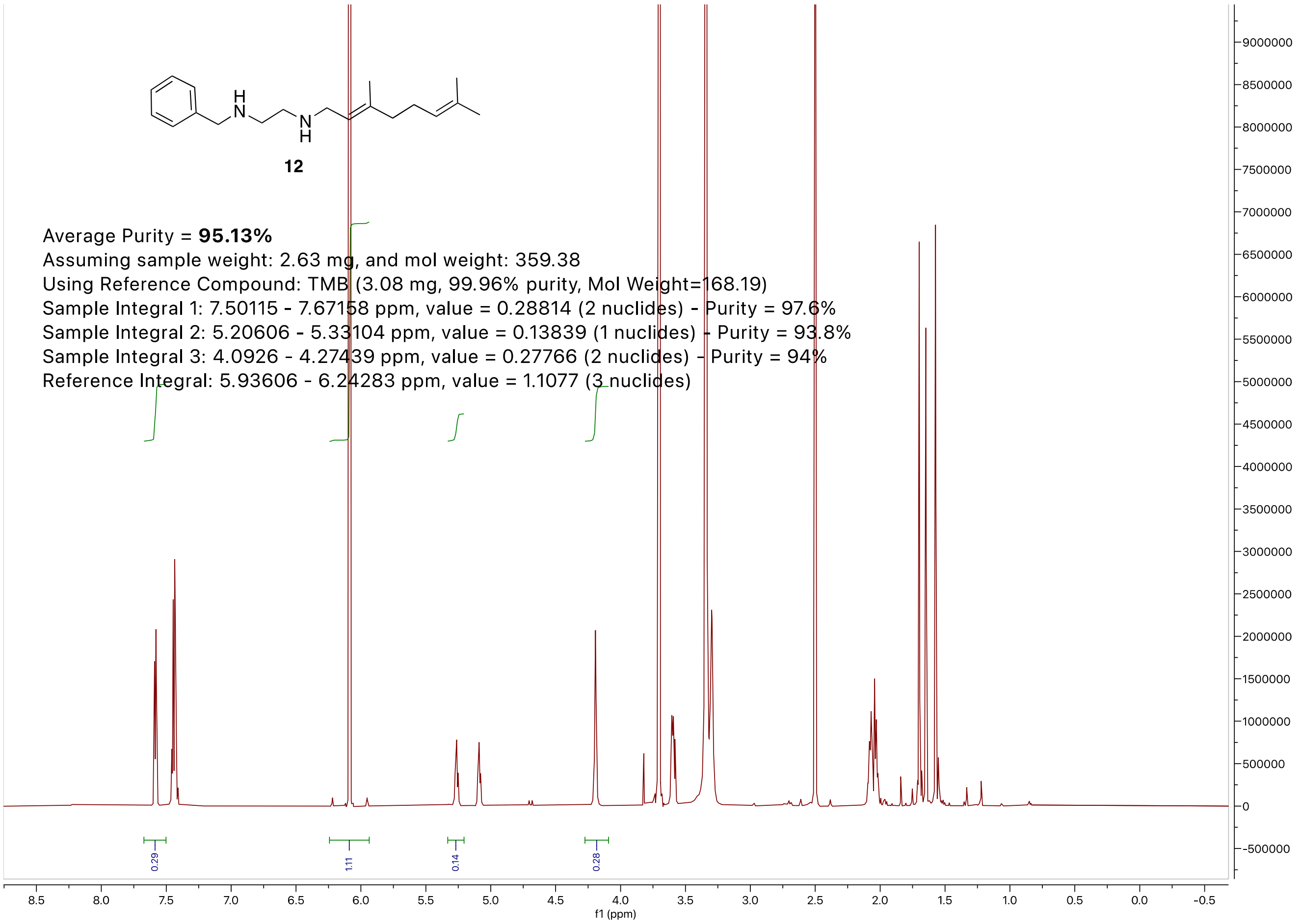
12
Average Purity = **95.13%**
Assuming sample weight: 2.63 mg, and mol weight: 359.38
Using Reference Compound: TMB (3.08 mg, 99.96% purity, Mol Weight=168.19)
Sample Integral 1: 7.50115 - 7.67158 ppm, value = 0.28814 (2 nuclides) - Purity = 97.6%
Sample Integral 2: 5.20606 - 5.33104 ppm, value = 0.13839 (1 nuclides) - Purity = 93.8%
Sample Integral 3: 4.0926 - 4.27439 ppm, value = 0.27766 (2 nuclides) - Purity = 94%
Reference Integral: 5.93606 - 6.24283 ppm, value = 1.1077 (3 nuclides)
f1 (ppm)

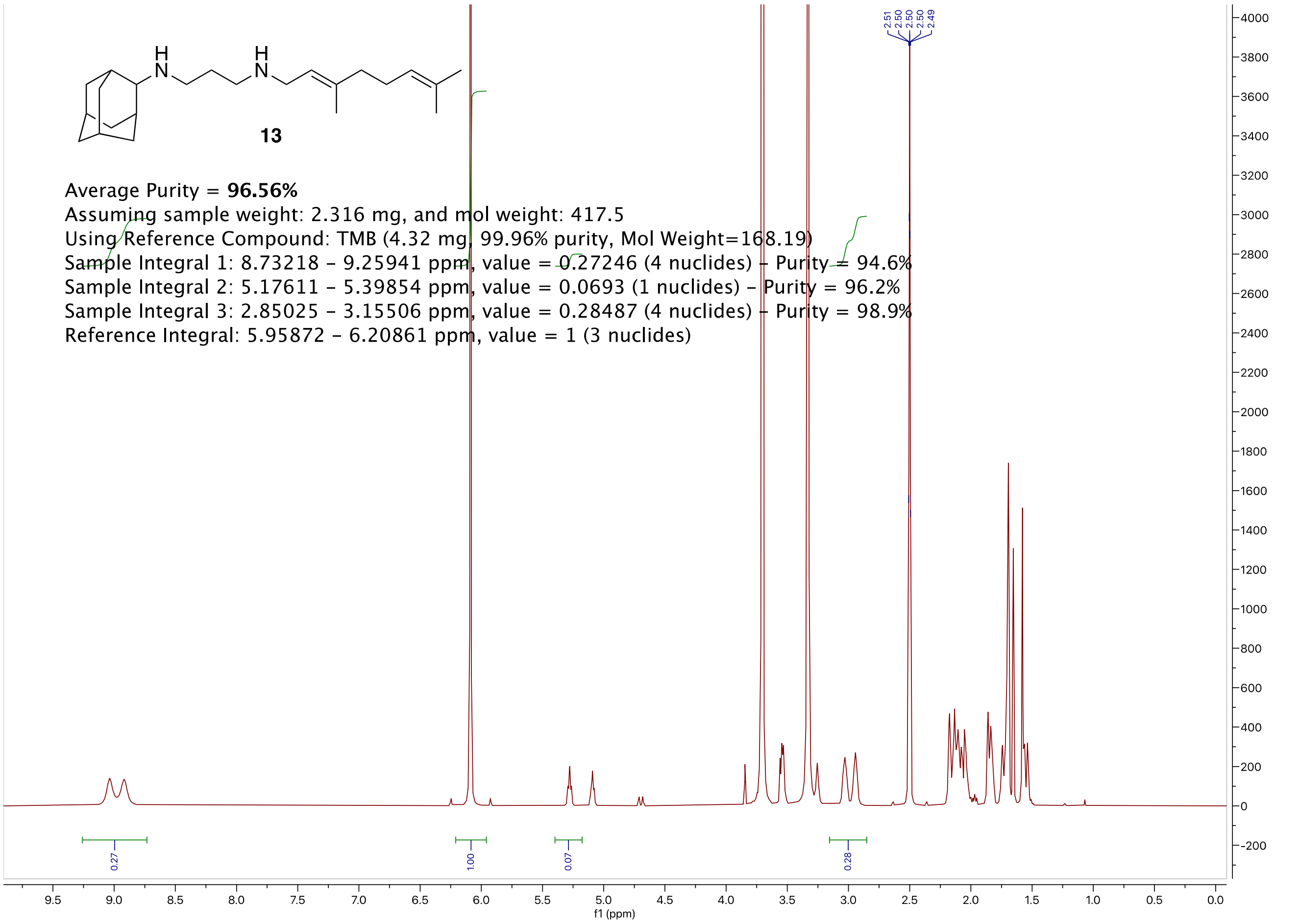
13
Average Purity = **96.56%**
Assuming sample weight: 2.316 mg, and mol weight: 417.5
Using Reference Compound: TMB (4.32 mg, 99.96% purity, Mol Weight=168.19)
Sample Integral 1: 8.73218 – 9.25941 ppm, value = 0.27246 (4 nuclides) – Purity = 94.6%
Sample Integral 2: 5.17611 – 5.39854 ppm, value = 0.0693 (1 nuclides) – Purity = 96.2%
Sample Integral 3: 2.85025 – 3.15506 ppm, value = 0.28487 (4 nuclides) – Purity = 98.9%
Reference Integral: 5.95872 – 6.20861 ppm, value = 1 (3 nuclides)
f1 (ppm)

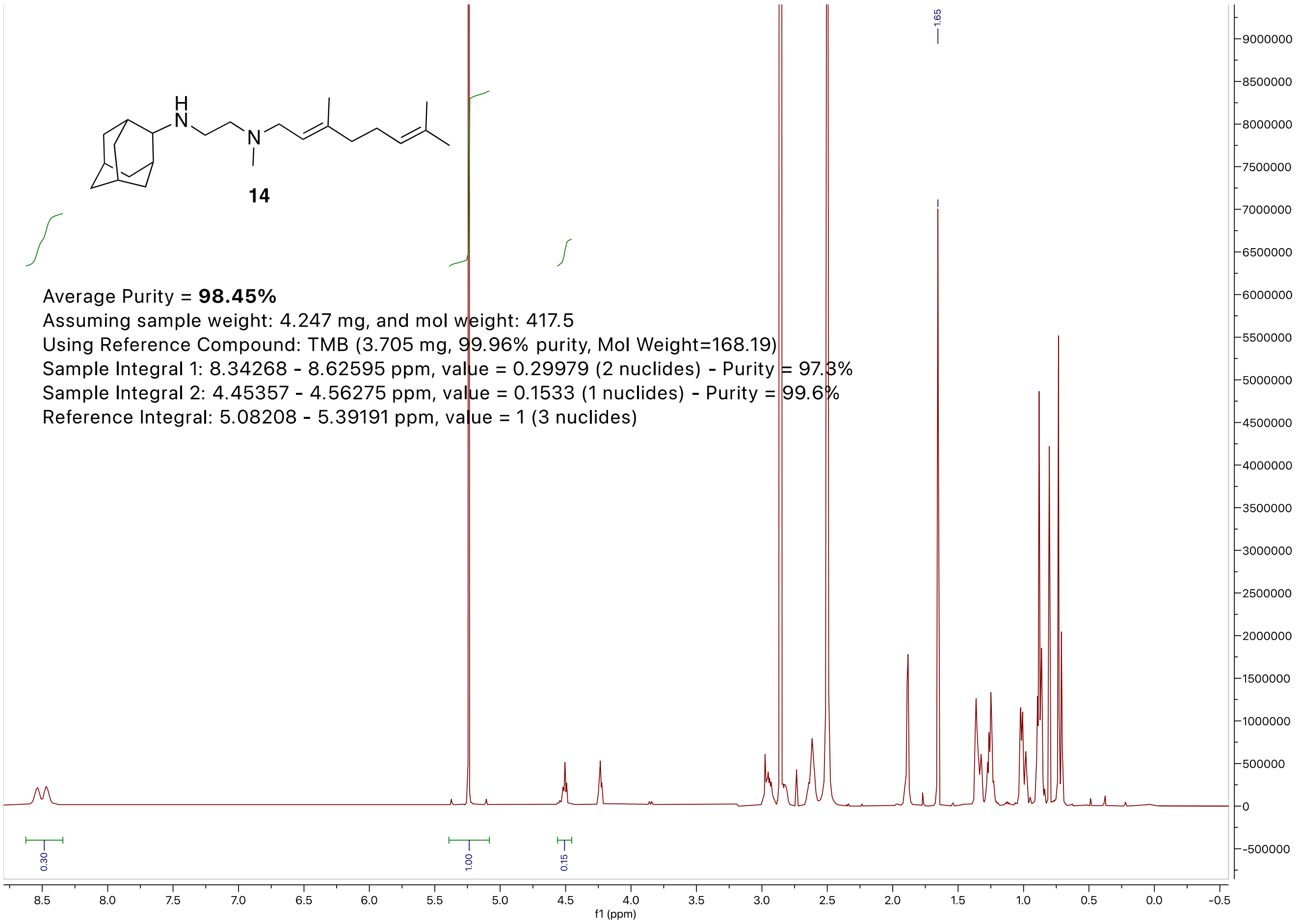
14
Average Purity = **98.45%**
Assuming sample weight: 4.247 mg, and mol weight: 417.5
Using Reference Compound: TMB (3.705 mg, 99.96% purity, Mol Weight=168.19)
Sample Integral 1: 8.34268 - 8.62595 ppm, value = 0.29979 (2 nuclides) - Purity = 97.3%
Sample Integral 2: 4.45357 - 4.56275 ppm, value = 0.1533 (1 nuclides) - Purity = 99.6%
Reference Integral: 5.08208 - 5.39191 ppm, value = 1 (3 nuclides)
f1 (ppm)

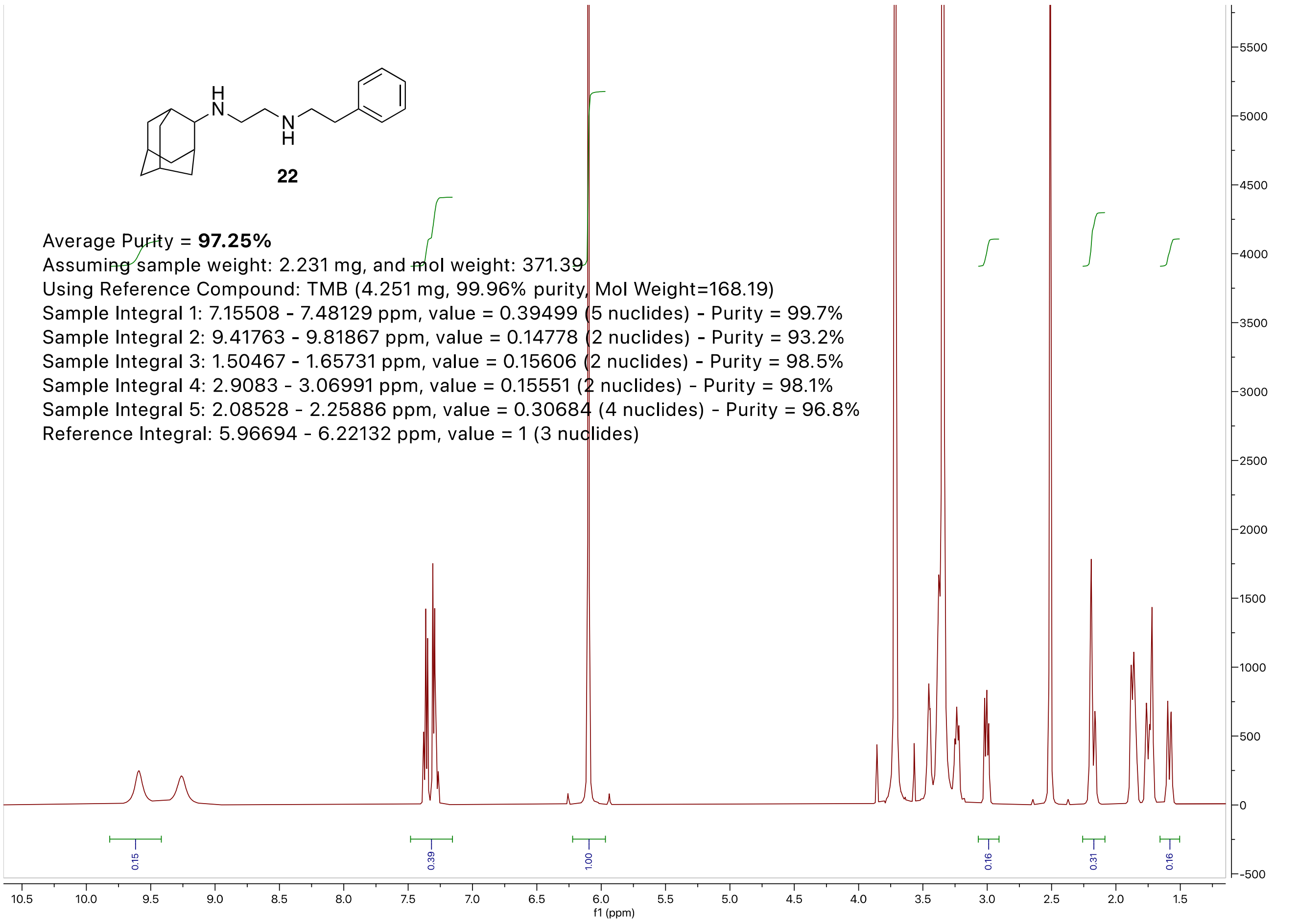
22
Average Purity = **97.25%**
Assuming sample weight: 2.231 mg, and mol weight: 371.39
Using Reference Compound: TMB (4.251 mg, 99.96% purity, Mol Weight=168.19)
Sample Integral 1: 7.15508 - 7.48129 ppm, value = 0.39499 (5 nuclides) - Purity = 99.7%
Sample Integral 2: 9.41763 - 9.81867 ppm, value = 0.14778 (2 nuclides) - Purity = 93.2%
Sample Integral 3: 1.50467 - 1.65731 ppm, value = 0.15606 (2 nuclides) - Purity = 98.5%
Sample Integral 4: 2.9083 - 3.06991 ppm, value = 0.15551 (2 nuclides) - Purity = 98.1%
Sample Integral 5: 2.08528 - 2.25886 ppm, value = 0.30684 (4 nuclides) - Purity = 96.8%
Reference Integral: 5.96694 - 6.22132 ppm, value = 1 (3 nuclides)
f1 (ppm)

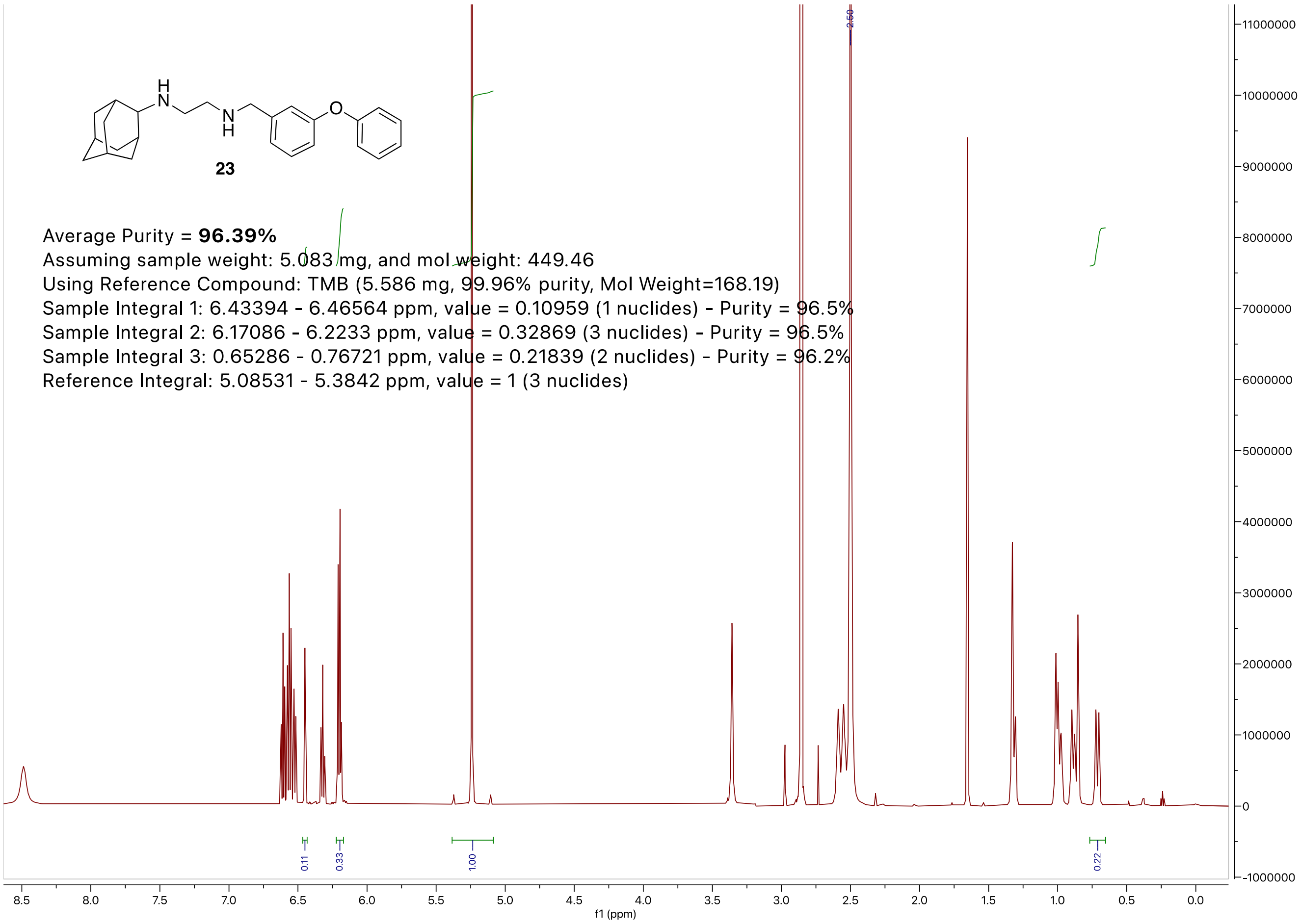
23
Average Purity = **96.39%**
Assuming sample weight: 5.083 mg, and mol weight: 449.46
Using Reference Compound: TMB (5.586 mg, 99.96% purity, Mol Weight=168.19)
Sample Integral 1: 6.43394 - 6.46564 ppm, value = 0.10959 (1 nuclides) - Purity = 96.5%
Sample Integral 2: 6.17086 - 6.2233 ppm, value = 0.32869 (3 nuclides) - Purity = 96.5%
Sample Integral 3: 0.65286 - 0.76721 ppm, value = 0.21839 (2 nuclides) - Purity = 96.2%
Reference Integral: 5.08531 - 5.3842 ppm, value = 1 (3 nuclides)
f1 (ppm)

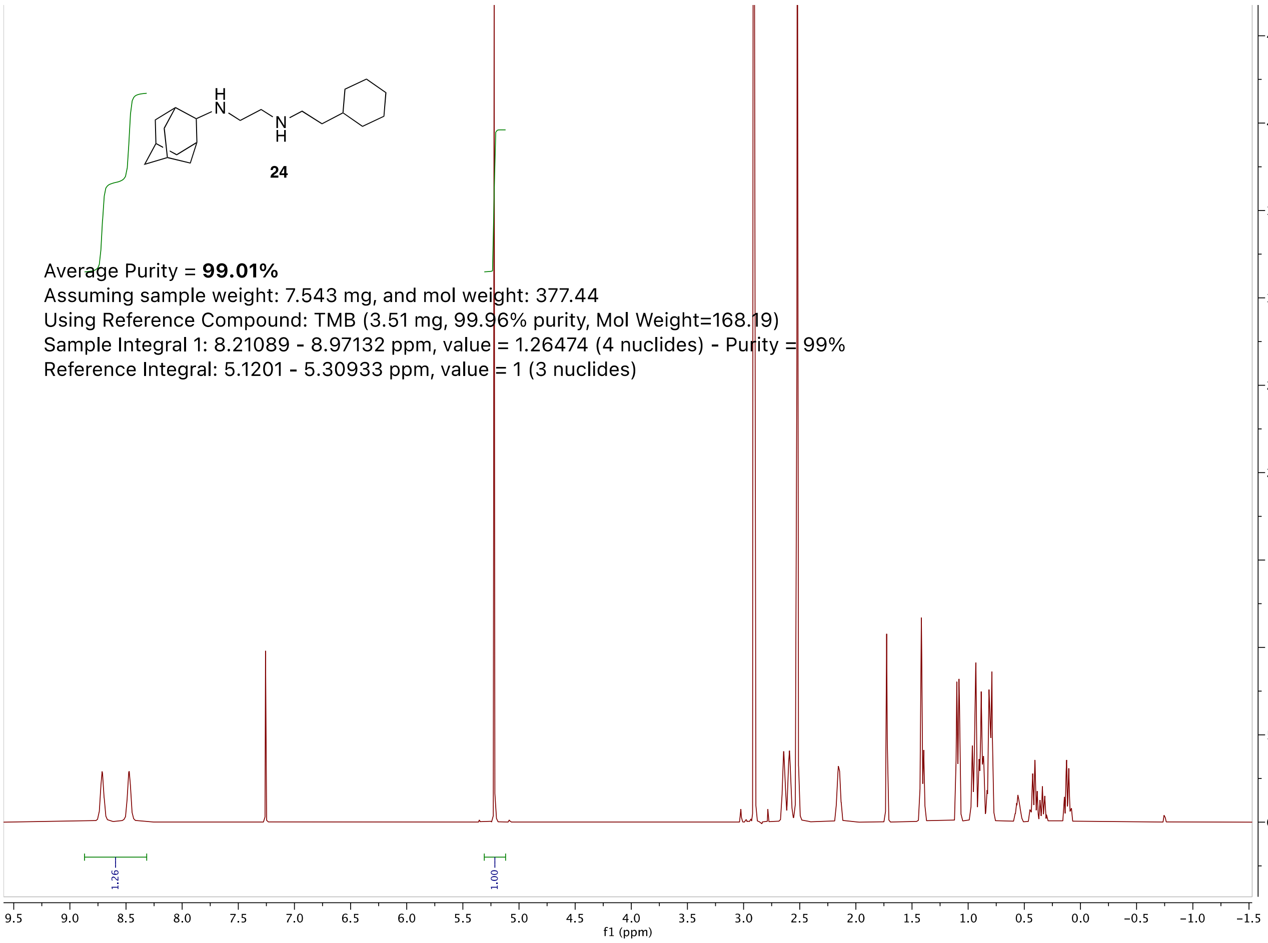
24
Average Purity = **99.01%**
Assuming sample weight: 7.543 mg, and mol weight: 377.44
Using Reference Compound: TMB (3.51 mg, 99.96% purity, Mol Weight=168.19)
Sample Integral 1: 8.21089 - 8.97132 ppm, value = 1.26474 (4 nuclides) - Purity = 99%
Reference Integral: 5.1201 - 5.30933 ppm, value = 1 (3 nuclides)
f1 (ppm)

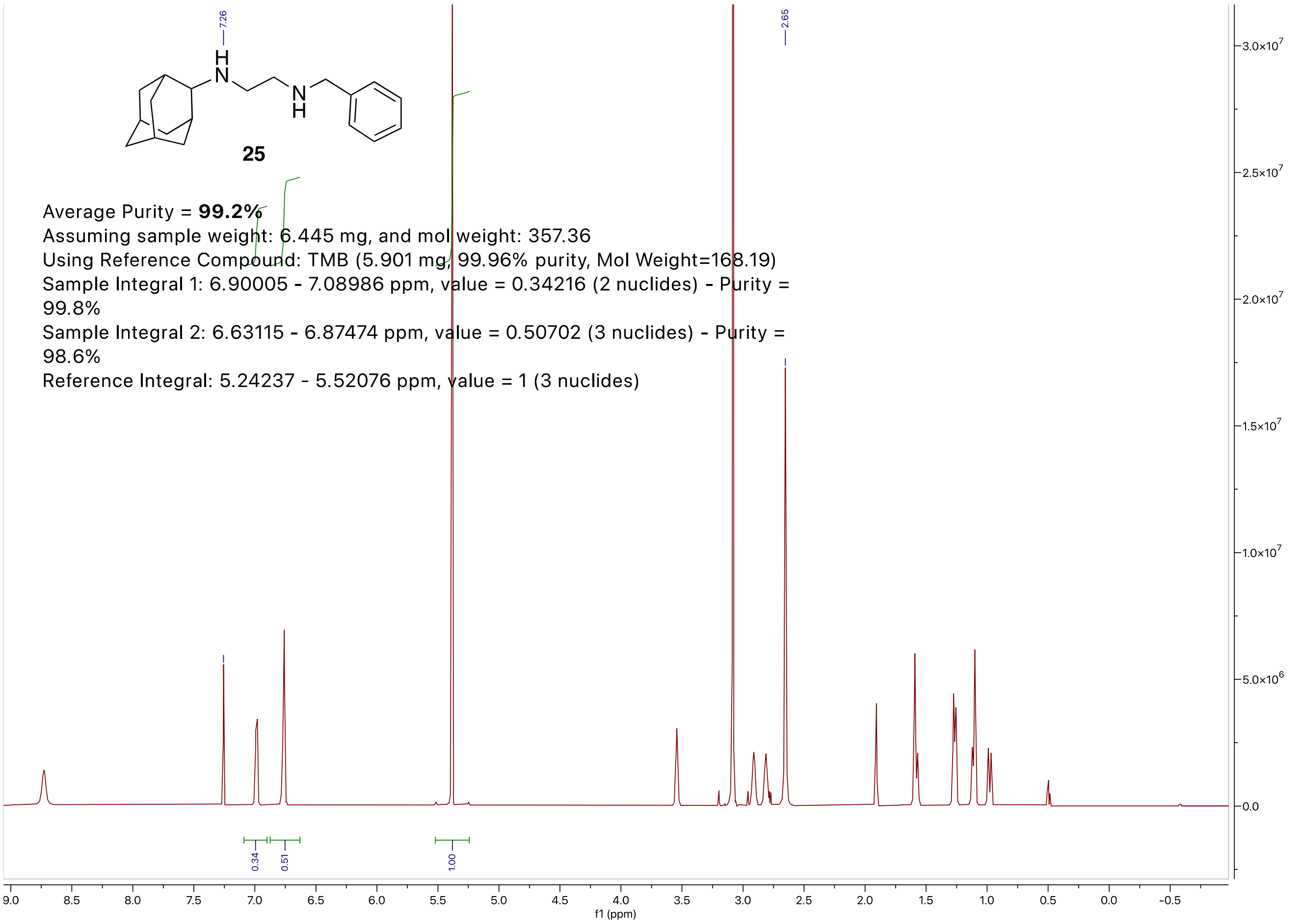

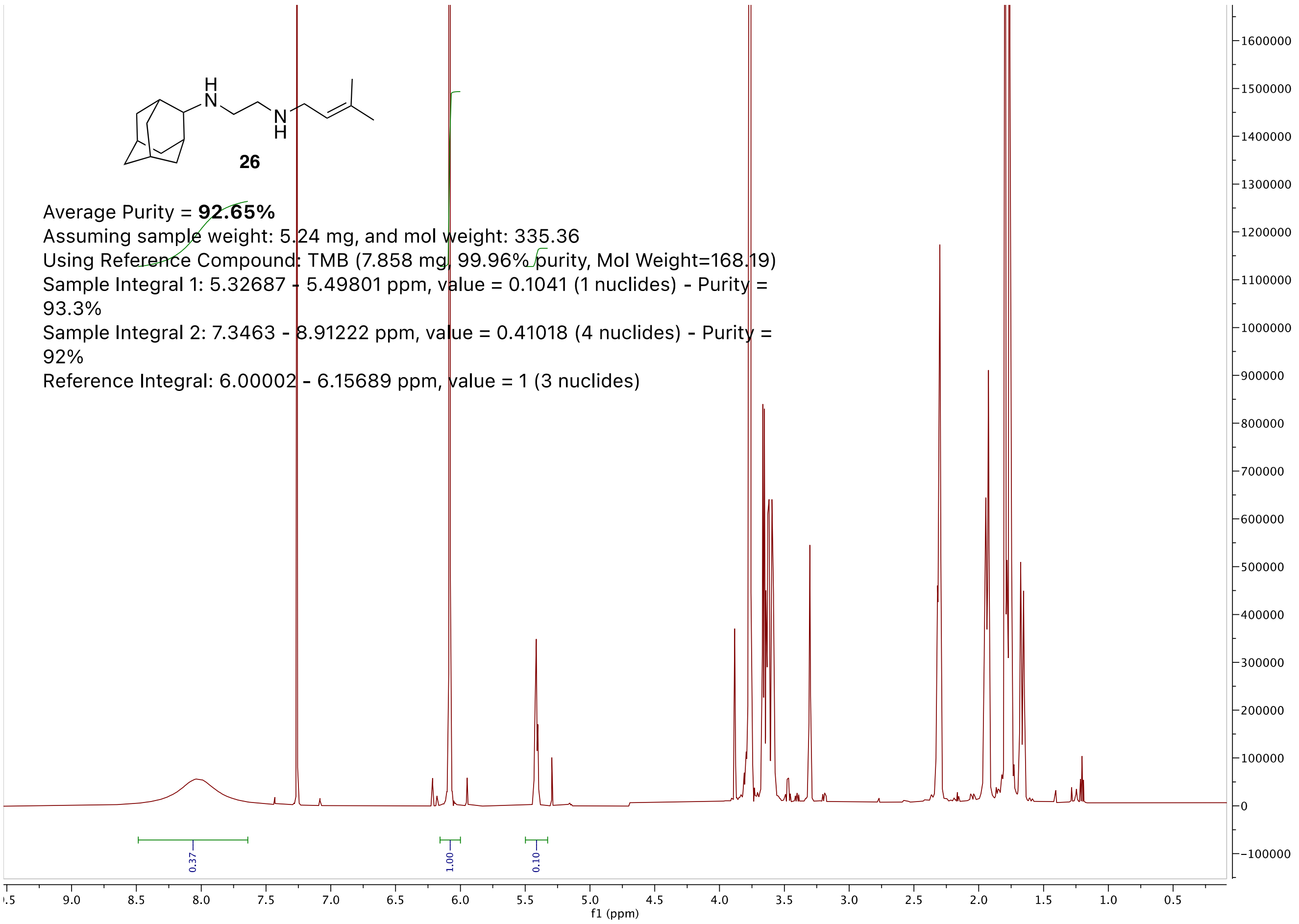

Figure S5. qNMR spectra and purity data for compounds 10-14; 22-26.

qNMR analysis of SQ109 **(1)**

Spectral Data Services, Inc.
818 Pioneer Street, Champaign, IL 61820
Phone: (217) 352-7084
Web: www.sdsnmr.com
Email: sdsnmr@sdsnmr.com

NMR Calculations

Oldfield Lab
12/1/2023

Calculations for wt-% purity via ^{1}H NMR
Sample: **SQ109**
Files: **oldze23002-004**

^{1}H NMR wt-% purity = (Ns/Ne) (MWe/MWs) (Ws/We) (Ie/Is) P

Ns = # of protons used for TMB (~6.1 ppm)		=	3
Ne = # of protons used for sample (~5.45 ppm)		=	1
MWs = Molecular weight of TMB		=	168.19 g/mol
MWe = Molecular weight of sample		=	403.48 g/mol
Ws = Weight of TMB	QS #1	=	10.3800 mg
	QS #2	=	10.6700 mg
	QS #3	=	16.5900 mg
We = Weight of sample	QS #1	=	11.15 mg
	QS #2	=	10.46 mg
	QS #3	=	12.49 mg
Is = Intensity of TMB protons used	QS #1	=	100.0000
	QS #2	=	100.0000
	QS #3	=	100.0000
Ie = Intensity of sample protons used	QS #1	=	14.8620
	QS #2	=	13.5234
	QS #3	=	10.3947
P = Purity of TMB Aldrich (Lot STBK3813)		=	99.95%

wt-% purity QS #1= 99.52%
wt-% purity QS #2= 99.23%
wt-% purity QS #3= 99.32%

Average wt-% purity = **99.36%** Standard Deviation (±) = **0.15%**

Analyst Signature: [signature] Date: 12/01/23

Reviewer Signature: [signature] Date: 12/1/23

REFERENCES
(1) Onajole, O. K.; Coovadia, Y.; Kruger, H. G.; Maguire, G. E.; Pillay, M.; Govender, T. Novel polycyclic 'cage'-1, 2-diamines as potential anti-tuberculosis agents. *European journal of medicinal chemistry* **2012**, *54*, 1-9.
(2) Onajole, O. K.; Govender, P.; van Helden, P. D.; Kruger, H. G.; Maguire, G. E.; Wiid, I.; Govender, T. Synthesis and evaluation of SQ109 analogues as potential anti-tuberculosis candidates. *European journal of medicinal chemistry* **2010**, *45* (5), 2075-2079.

Compd #	SMILES
**1**	C/C(C)=C/CC/C(C)=C/CNCCNC1[C@@H]2C[C@H](C[C@H]1C3)C[C@H]3C2
**2**	C/C(C)=C/CC/C(C)=C/CNCCNC1(CC)[C@@H]2C[C@H](C[C@H]1C3)C[C@H]3C2
**3**	C/C(C)=C/CC/C(C)=C/CNCCNC1(CCCC)[C@@H]2C[C@H](C[C@H]1C3)C[C@H]3C2
**4**	C/C(C)=C/CC/C(C)=C/CNCCNC1(CC2=CC=CC=C2)[C@@H]3C[C@H](C[C@H]1C4)C[C@H]4C3
**5**	C/C(C)=C/CC/C(C)=C/CNCCNC1(C2=CC=CC=C2)[C@@H]3C[C@H](C[C@H]1C4)C[C@H]4C3
**6**	C/C(C)=C/CC/C(C)=C/CNCCNC1(CCC)[C@@H]2C[C@H](C[C@H]1C3)C[C@H]3C2
**7**	C/C(C)=C/CC/C(C)=C/CNCCNC1(C)[C@@H]2C[C@H](C[C@H]1C3)C[C@H]3C2
**8**	CC(C)(NCCNC/C=C(C)/CC/C=C(C)/C)C1[C@@H]2C[C@H](C[C@H]1C3)C[C@H]3C2
**9**	C/C(C)=C/CC/C(C)=C/CNCCNC12C[C@@H]3C[C@H](C1)C[C@H](C2)C3
**10**	C/C(C)=C/CC/C(C)=C/CNCCNC1CC2CC1CC2
**11**	C/C(C)=C/CC/C(C)=C/CNCCNC1CC2CCC1CC2
**12**	C/C(C)=C/CC/C(C)=C/CNCCNCC1=CC=CC=C1
**13**	C/C(CC/C=C(C)/C)=C\CNCCCNC1[C@@H]2C[C@H](C[C@H]1C3)C[C@H]3C2
**14**	C/C(C)=C/CC/C(C)=C/CN(C)CCNC1[C@@H]2C[C@H](C[C@H]1C3)C[C@H]3C2
**15**	C/C(C)=C/CC/C(C)=C/COCCNC1[C@@H]2C[C@H](C[C@H]1C3)C[C@H]3C2
**16**	C/C(C)=C/CC/C(C)=C/CNCCOC1[C@@H]2C[C@H](C[C@H]1C3)C[C@H]3C2
**17**	C/C(C)=C/CC/C(C)=C/CNCC(NC1[C@@H]2C[C@H](C[C@H]1C3)C[C@H]3C2)=O
**18**	C/C(C)=C/CC/C(C)=C/CNC(CNC1[C@@H]2C[C@H](C[C@H]1C3)C[C@H]3C2)=O
**19**	CC(C)CCCC(C)CCNCCNC1[C@@H]2C[C@H](C[C@H]1C3)C[C@H]3C2
**20**	CC(C)CCCC(C)CCCC(C)CCNCCNC1[C@@H]2C[C@H](C[C@H]1C3)C[C@H]3C2
**21**	C/C(C)=C/CC/C(C)=C/CC/C(C)=C/CNCCNC1[C@@H]2C[C@H](C[C@H]1C3)C[C@H]3C2
**22**	[C@@H]1(C[C@@H]2C3NCCNCCC4=CC=CC=C4)C[C@H](C2)C[C@H]3C1
**23**	[C@@H]1(C[C@@H]2C3NCCNCC4=CC=CC(OC5=CC=CC=C5)=C4)C[C@H](C2)C[C@H]3C1

**24**	[C@@H]1(C[C@@H]2C3NCCNCCC4CCCCC4)C[C@H](C2)C[C@H]3C1
**25**	[C@@H]1(C[C@@H]2C3NCCNCC4=CC=CC=C4)C[C@H](C2)C[C@H]3C1
**26**	C/C(C)=C/CNCCNC1[C@@H]2C[C@H](C[C@H]1C3)C[C@H]3C2
